# Ethnobotanical and ethnomedicinal research into medicinal plants in the Mt Stara Planina region (south-eastern Serbia, Western Balkans)

**DOI:** 10.1186/s13002-024-00647-2

**Published:** 2024-01-10

**Authors:** Snežana Jarić, Olga Kostić, Zorana Miletić, Milica Marković, Dimitrije Sekulić, Miroslava Mitrović, Pavle Pavlović

**Affiliations:** Department of Ecology, Institute for Biological Research ‘Siniša Stanković’ - National Institute of the Republic of Serbia, University of Belgrade, Bulevar Despota Stefana 142, 11108 Belgrade, Serbia

**Keywords:** Ethnobotany, Ethnomedicine, Mt Stara Planina, Medicinal plant diversity, Traditional knowledge

## Abstract

**Background:**

Ethnobotanical research in Southeast Europe—one of the most important European hotspots for biocultural diversity—is significant for the acquisition of Traditional Ecological Knowledge related to plants as well as for encouraging the development of local environments. The current ethnobotanical research was conducted in the region of Mt Stara Planina (south-eastern Serbia), which is characterised by rich phytodiversity with a large number of endemic and relict plant species. The aim of the study was to document the diversity of uses of medicinal plants and of traditional knowledge on their therapeutic uses.

**Methods:**

Ethnobotanical data was collected through both open and semi-structured interviews with locals. Fifty-one inhabitants were interviewed (26 men and 25 women), aged 30–91, and data was analysed by means of use reports, citation frequency, use values (UV), and the informant consensus factor (ICF).

**Results:**

The study identified 136 vascular medicinal plant taxa and one lichen species belonging to 53 families and 116 genera. Lamiaceae (19), Rosaceae (18), and Asteraceae (17) had the highest species diversity. The plant parts most commonly used to make a variety of herbal preparations were the aerial parts (54 citations), leaves (35 citations), fruits (20 citations), flowers (18 citations), and roots (16 citations), while the most common forms of preparation were teas (60.78%), consumption of fresh tubers, leaves, roots, and fructus (6.86%), compresses (5.88%), juices (5.39%), decoctions (3.92%), ‘travarica’ brandy (3.92%), and syrups (2.45%). Of the recorded species, 102 were administered orally, 17 topically, and 18 both orally and topically. The plants with a maximum use value (UV = 1) were *Allium sativum, Allium ursinum, Gentiana asclepiadea, Gentiana cruciata, Gentiana lutea, Hypericum perforatum, Thymus serpyllum* and *Urtica dioica*. The highest ICF value (ICF = 0.95) was recorded in the categories of Skin and Blood, Blood Forming Organs, and Immune Mechanism.

**Conclusions:**

This study shows that medicinal plants in the research area are an extremely important natural resource for the local population as they are an important component of their health culture and provide a better standard of living.

## Background

Europe has a long history of the transmission of medical knowledge despite its geographical, cultural, and linguistic barriers [1]. Rural regions in some parts of Europe, such as the mountainous regions of the Balkan Peninsula, are unique hotspots of biological and cultural diversity. As such they are considered highly significant for the conducting of studies with a human ecological focus, thus allowing cross-cultural comparisons of traditional ecological knowledge concerning medicinal plants [2–16]. Ethnobotanical studies are mainly conducted with the indigenous peoples in local communities where traditional knowledge is scarcely documented. This is mainly due to the way the information is passed down, mostly orally, from generation to generation. Also, an alarming rate of decline in traditional medical knowledge in the region of Southeast Europe is a result of major political turmoil and economic changes that have greatly affected lifestyles, eating habits, and relationships with nature, as well as the transfer of traditional knowledge on health and local medical practices [17]. For this reason, the role of traditional knowledge, indigenous communities, and ethnobotanists in achieving sustainable development goals has to be recognised as a matter of urgency [18]. It is of the utmost importance that plant biodiversity be preserved so as to provide future structural diversity and important medical components for the sustainable development of human populations worldwide. Well-planned bioprospecting coupled with non-destructive commercialisation could help in the conservation of biodiversity, ultimately benefiting humankind in the long run [19].

Among indigenous people, particularly in the rural regions of the Balkan Peninsula including Serbia, there is a strong belief in the power of medicinal plants, which is linked to traditions originating over the past centuries. The sound knowledge of ethnobotany in these areas is also a result of the specific geographical position, great biological diversity, and ethnic and cultural differences.

In regard to this, we conducted ethnobotanical research focused on the use of medicinal plants in the Mt Stara Planina region, which is located in south-eastern Serbia, in the Western Balkans. This area is characterised by high diversity of plant and animal species and their communities, as well as of geomorphological, geological, hydrological, and hydrogeological phenomena. The traditional way of life is still found here and it abounds in cultural heritage [20]. It has also been designated as internationally significant for plants (important plant areas—IPA), diurnal butterflies (primary butterfly areas—PBA), and birds (internationally important bird areas—IBA) and is on the list of Serbian geo-heritage sites (International Association for the Conservation of Geological Heritage—ProGEO) [21].

Knowledge of the diversity and use of medicinal plants, especially in rural areas like the Mt Stara Planina region, enables the development of modern ecologically organised human activities (ecological management, sustainable development, ecotourism, ecological engineering), which improves the living conditions of the local inhabitants and contributes to maintaining the ecological balance in nature. Historically, the Balkan region has provided plant material for the West European market, especially in the last few centuries. Most locally harvested medicinal plants, in dry or raw form, are widely used in local health care in many households and they are at the heart of many economic initiatives and programmes dedicated to rural development [17]. The increased volume for trade and increased demand for certain types of medicinal plants has resulted in the need for sustainable management of these natural resources based on biodiversity conservation strategies [22]. In this regard, previous or current ethnobotanical studies in Southeast Europe, where the study area is located, are of vital importance for stimulating local development and investigating the dynamics of Traditional Ecological Knowledge (TEK), which relates to plants as an important natural resource in one of the most crucial European hotspots for biocultural diversity [9]. Wild medicinal plants are an important commercial product (in the form of raw or dried plant material), but they are primarily a natural resource that should be used wisely and rationally. Therefore, the objectives of this study were: (1) to identify plant species traditionally used for the treatment and prevention of various health conditions and diseases among the local population, (2) to distinguish plant species with a maximum use value (UV), (3) to determine consensus levels among the informants by using the informant consensus factor (ICF), (4) to report on new species data and new usage data not previously recorded for Serbia and the Balkans, and (5) to discuss the significance of these medicinal plants for the herbal market, as foodstuffs, and for ecotourism.

These studies can contribute to the general fund of traditional ethnobotanical and ethnomedical knowledge in the Western Balkans and beyond and to the promotion of the practical importance of the use of medicinal plants and their conservation. This type of research can contribute to the conservation of biodiversity and the sustainable use of medicinal plants in order to develop the economy of the local population.

## Methods

### Study area

**Fig. 1:**
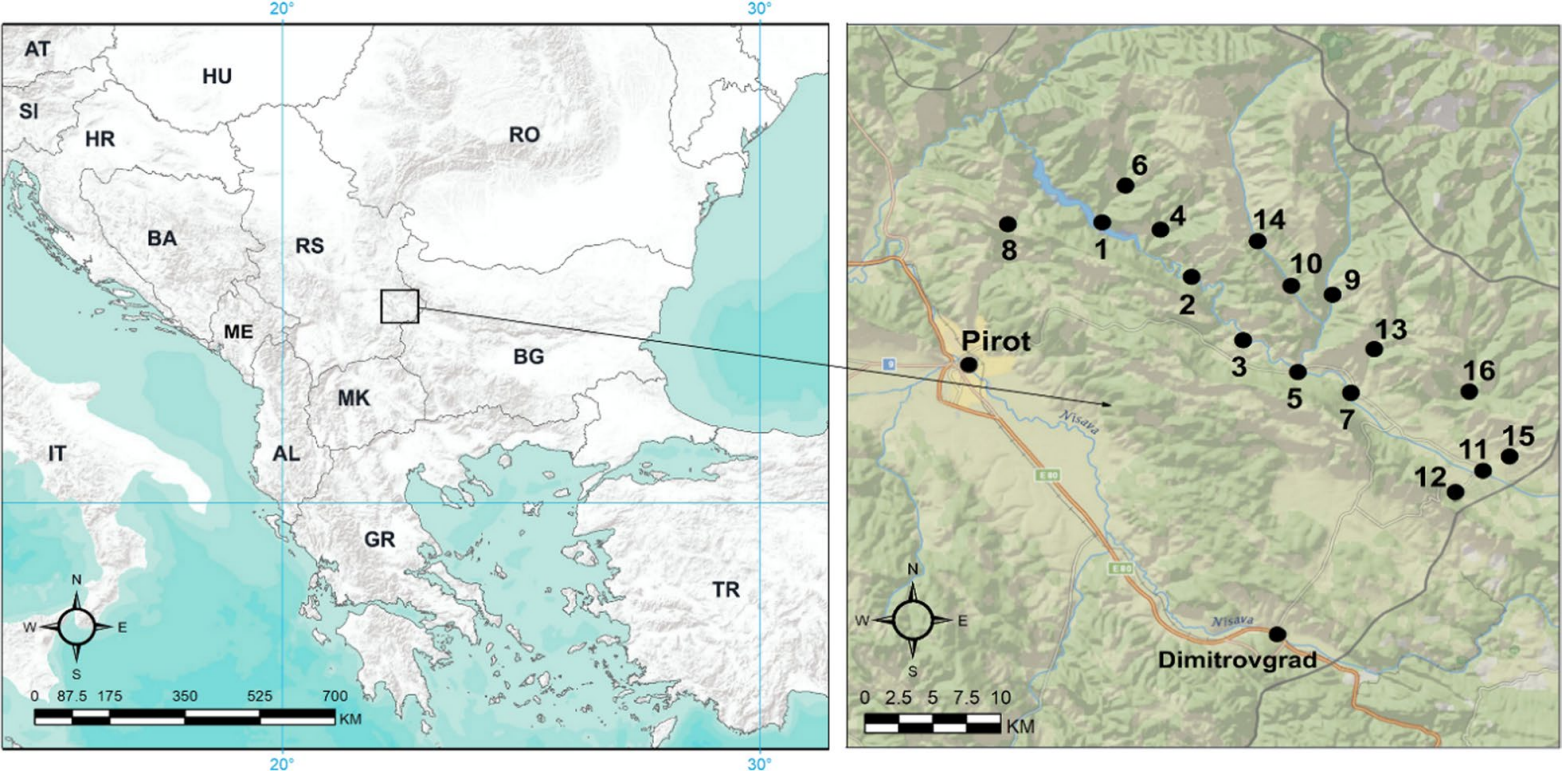
Map of the study area. Investigated localities: 1. Velika Lukanja; 2. Pakleštica; 3. Rsovci; 4. Bela; 5. Visočka Ržana; 6. Gostuša; 7. Slavinja; 8. Koprivštica; 9. Jelovica; 10. Brlog; 11. Donji Krivodol; 12. Vlkovija; 13. Rosomač; 14. Dojkinci; 15. Gornji Krivodol 16. Senokos

Stara Planina Mountains or the Balkan Mountains are a mountain range mainly located in Bulgaria with a much smaller part in south-eastern Serbia. It is part of the Carpathian–Balkan mountain arc and extends from the Black Sea in Bulgaria in the east to Vrška Čuka (Zaječar, Serbia) in the west, with a total length of 530 km. A small section of this range is located in the eastern part of Serbia, lying in the municipalities of Zaječar, Knjaževac, Pirot, and Dimitrovgrad, and as a morphological unit it is bound by the valleys of the Beli Timok, Trgoviški Timok, and Visočica rivers and the state border to the east [23] (Fig. 1). The highest point of the Stara Planina mountain range in the region of south-eastern Serbia, where our research was conducted, is the peak of Midžor (2169 m a.s.l.), while the lowest is at the exit of the Prlitski Potok valley (132 m a.s.l.). In terms of climate, Mt Stara Planina is a subregion of Eastern Serbia, which is impacted primarily by the Vlach-Pontic Basin, but also by the Pannonian and, to a much lesser extent, the Aegean Basins. The prevailing climate at the base of the mountains is continental [24]. The climatic elements of Mt Stara Planina exhibit significant spatial variability in terms of their mean values due to the high altitude gradient (up to 2000 m), the direction the main mountain range extends in, and its relief, which sees frequent and large changes in the gradient and exposure of mountain slopes and valley sides. Average temperatures decrease and humidity increases with increase in altitude. The coldest month of the year is January with an average temperature of 0.5 °C, while the hottest month is July with an average temperature of 22 °C. Average annual precipitation is 960.5 mm with the highest levels in May and the lowest in September [25].

The relief of this area is fundamentally tectonic in origin (morphostructures formed by the effects of tectonic movements) and is shaped by eluvial, diluvial, proluvial, colluvial, fluvial, and karst processes [26]. The existence of different types of soils on Mt Stara Planina is the result of the specificity of the geological substrate and the prevailing climatic conditions, with rankers, rendzinas, vertisols, cambisols, and recent soils present, while wetlands have developed in places with high groundwater levels [26, 27]. Moreover, the hydrography of this area is complex and features aquifers, springs, streams, and mountain rivers. The spring with the coldest water (4 °C) in Serbia is found below the peak known as ‘Vražja glava’ [28].

### The diversity of flora and vegetation

The vascular flora of the Stara Planina mountain range (south-eastern Serbia) consists of 1742 taxa (species and subspecies), which is why this area is classified as having the highest floristic diversity and density of flora per unit area in Europe [20]. The vertical zonation of vegetation, caused by the morphological differentiation of the terrain, large differences in altitude (132–2169 m), and the climatic characteristics, is extremely pronounced. There are five clear altitudinal vegetation zones in terms of the vertical profile, reflecting the climatic, geomorphological, geological, and edaphic characteristics of Mt Stara Planina: oak, beech, spruce, subalpine, and alpine [20, 26]. Mišić et al. [26] described a total of 52 phytocoenoses (24 forest and shrub and 28 herbaceous) in this area, with 160 endemic and subendemic species, which is 9.12% of the total flora of this mountain range [20]. There are also seven strict nature reserves on this mountain: ‘Draganište’, ‘Golema reka’, ‘Vražja glava’, ‘Tri čuke’, ‘Arbinje’, ‘Bratkova strana’, and ‘Kopren’.

Due to the exceptional diversity of the flora and fauna, as well as its geomorphological, geological, hydrological, and hydrogeological features, a 142,219.64 ha area of the Mt Stara Planina region was proclaimed a Nature Park in 1997. Later, in 2009, a Decree was adopted at the national level, designating the ‘Stara Planina’ Nature Park a protected natural resource of exceptional importance, i.e. Category 1 status (Official Gazette of the Republic of Serbia, no. 23/09).

### Data collection and analysis

Ethnobotanical research was conducted between 2020 and 2022 in 16 villages: (1) Mala Lukanja, (2) Gostuša, (3) Velika Lukanja, (4) Bela, (5) Pakleštica, (6) Dojkinci, (7) Brlog, (8) Jelovica, (9) Rsovci, (10) Visočka Ržana, (11) Rosomač, (12) Slavinja, (13) Prelesje, (14) Senokos, (15) Gornji Krivodol, and (16) Donji Krivodol (Fig. 1; Table 1). The villages are relatively small in terms of area and particularly in terms of population. They are located at altitudes ranging from 612 to 895 m a.s.l.

**Table 1:** Characteristics of the investigated localities in the study area (coordinates, altitude, number of inhabitants, and ethnic composition)

No.	Locality	Latitude	Longitude	Altitude (m)	Number of inhabitants [31]	Ethnic composition
1	Velika Lukanja	43.23614	22.680532	612	6	Serbs
2	Pakleštica	43.206651	22.740408	628	32	Serbs
3	Rsovci	43.1723509	22.775029	671	106	Serbs
4	Bela	43.232228	22.719701	677	24	Serbs
5	Visočka Ržana	43.1550308	22.8116251	704	23	Serbs
6	Gostuša	43.256123	22.6961582	705	70	Serbs
7	Slavinja	43.1439326	22.8471248	733	39	Serbs
8	Koprivštica	43.2351801	22.6174646	780	45	Serbs
9	Jelovica	43.197008	22.834923	791	87	Serbs, Roma, Bulgarians
10	Brlog	43.201841	22.807281	801	56	Serbs
11	Donji Krivodol	43.1016389	22.9357898	814	11	Bulgarians predominantly, Serbs
12	Vlkovija	43.0901484	22.9172164	824	17	Bulgarians
13	Rosomač	43.1675777	22.8628552	862	37	Serbs
14	Dojkinci	43.2260036	22.784642	868	176	Serbs
15	Gornji Krivodol	43.1094963	22.9535559	870	8	Bulgarians, Serbs
16	Senokos	43.144652	22.926472	895	29	Bulgarians (66%), Serbs

The population of the study area belongs to a particular variety of ethnic group with ancient Slavic origins known as the ‘Shopi’ or ‘Torlaks’. In terms of language, this group has managed to preserve its own dialect—the Timok-Luznica dialect with elements of Serbian, Bulgarian, and Turkish [29, 30]—while the official language of the region is Serbian. According to the 2011 census, inhabitants in the study area identify as Serbs, Bulgarians, and Roma. Bulgarians were in the majority in the villages of Senokos, Vlkovija, Gornji Krivodol, and Donji Krivodol, while Serbs predominated in the other villages (Table 1). In terms of religion, all the interviewees were Orthodox Christians.

Ethnobotanical data on the knowledge of plants and their use for medicinal purposes was collected using semi-structured and structured face-to-face interviews, conducted in Serbian, with inhabitants of the research area on several trips to this region between 2020 and 2022. The main criteria for selecting the interview participants were: the age of the respondents (those chosen were over 50 because it was assumed that they had ‘life’ experience and had at some point used a plant to treat an ailment), the participation of respondents of both sexes, and their residing permanently in the villages where we conducted the research. We interviewed 26 men and 25 women (*n *= 51), aged 30–91. The majority of the interviewees were aged between 51 and 70 (50.98%), while only two (3.92%) were older than 91 (Table 2). For all the respondents, we recorded their gender, age, level of education, and occupation. The majority had completed secondary education (54.90%), while traditional knowledge on the use of medicinal plants in the study area was collected from farmers (35.29%), housewives (27.45%), medicinal plant pickers (15.69%), and pensioners (21.57%).

**Table 2:** Demographic features of the local informants in the study area (*n *= 51)

Demographic features		Abundance	Relative abundance (%)
Gender	Male	26	50.98
	Female	25	49.02
Age group	30–50	3	5.88
	51–70	26	50.98
	71–90	20	39.22
	> 91	2	3.92
Education	Literate	2	3.92
	Primary level	21	41.18
	Secondary level	28	54.90
Occupations	Farmer	18	35.29
	Housewife	14	27.45
	Collector of medicinal plants	8	15.69
	Pensioner	11	21.57

In Serbia, there are no special rules or regulations pertaining to conducting ethnobotanical research. Before beginning each interview, the purpose, methodology, and nature of the research were explained to all participants, after which voluntary oral consent was obtained from all informants. Moreover, all of the respondents gave their oral consent for their photographs to be taken during the interview and published. Each informant had the chance to end the interview at any time. After the interviews, all the data was deposited at the Department of Ecology of the Institute for Biological Research 'Siniša Stanković', a National Institute of the Republic of Serbia. Ethnobotanical and ethnomedical research was conducted in accordance with the International Society of Ethnobiology (ISE) Code of Ethics [32]. All principles of the Code of Ethics were respected and there were no harmful consequences for the local community. Furthermore, all the recommended standards for conducting ethnobotanical research according to Weckerle et al. [33].

**Fig. 2:**
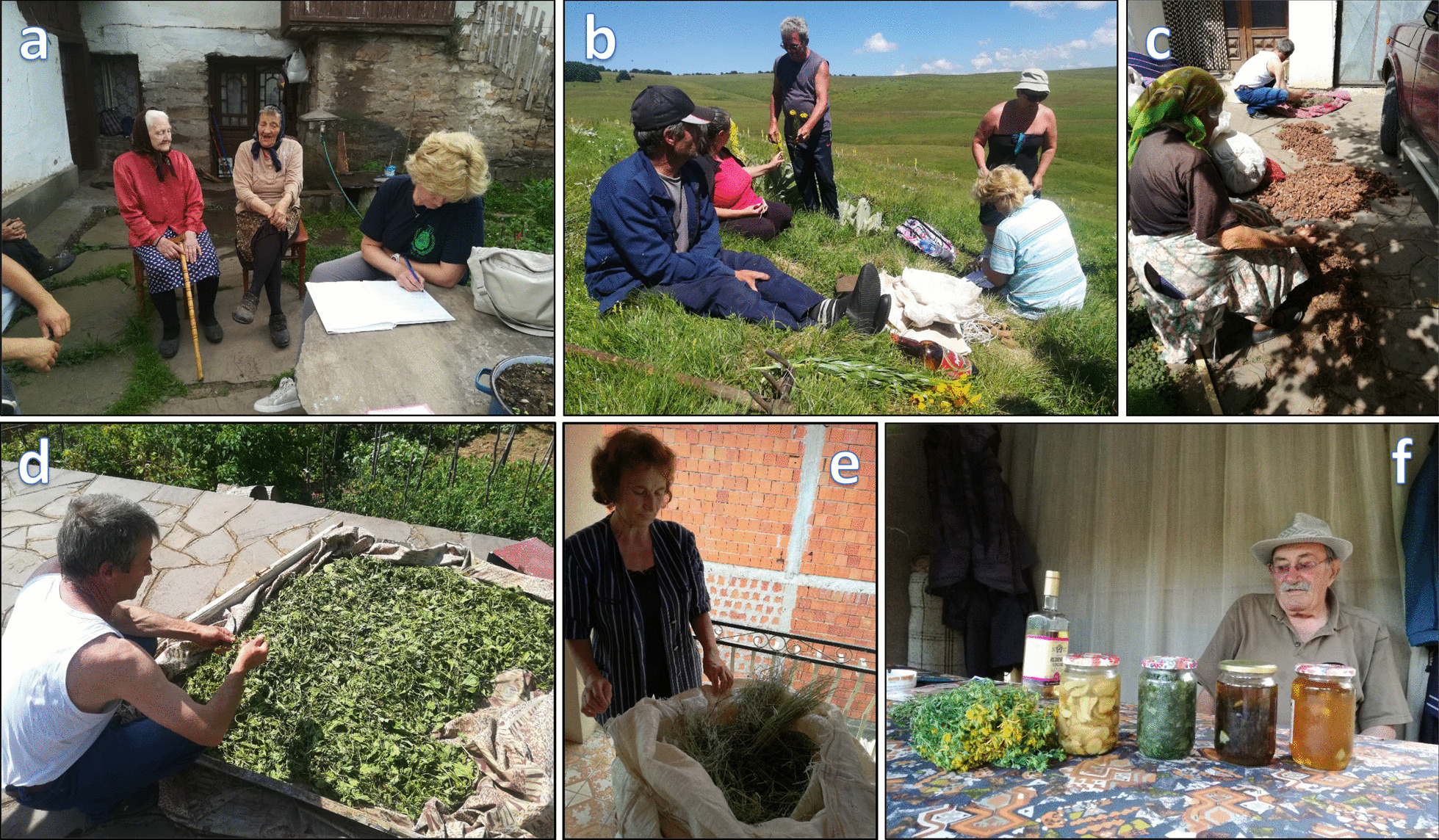
Field work **a,b** Data collection from local informants; **c–f** Methods of preparation of medicinal plants for further use

The focus of the interview was the local population’s traditional knowledge on the use of wild plants to treat various health problems (Fig. 2). One questionnaire was completed for each plant species mentioned as being used by the respondents. Identification of plants was performed in the field or from herbarium specimens using literature sources [34–36]. The correct taxonomy and nomenclature of the vascular plants was also checked referring to World Flora Online (WFO) (www.worldfloraonline.org), while for *Cetraria islandica* (L.) Ach. (Iceland lichen) the LIAS names web interface was used [37], (https://liasnames.lias.net/). Voucher specimens were deposited at the herbarium of the Institute for Biological Research ‘Siniša Stanković’—National Institute of the Republic of Serbia (IBISS) in Belgrade.

The International Classification of Primary Care (ICPC-2) of the World Health Organization (WHO) was used for the categorisation of all the diseases and ailments treated [38, 39]. In this study, the diseases and health problems which were mentioned by the respondents were classified into 16 categories: (1) Blood, Blood Forming Organs, and Immune Mechanism, (2) Respiratory, (3) General and Unspecified, (4) Digestive, (5) Cardiovascular, (6) Endocrine/Metabolic and Nutritional, (7) Ear, (8) Eye, (9) Psychological, (10) Urological, (11) Female Genital, (12) Male Genital, (13) Pregnancy, Childbearing, Family Planning, (14) Skin, (15) Musculoskeletal, and (16) Neurological.

MS Excel (2016) was used for data entry and summary. As part of the quantitative data analysis, the number of citations for each of the mentioned health problems was calculated as well as the informant consensus factor (ICF) as an index used to test the homogeneity of the knowledge shared by the informants and the UV of the plants which the respondents mentioned.

The informant consensus factor is determined using the formula: ICF = Nur − Nt/Nur − 1, where Nur refers to the total number of reports (number of mentions) on use for each disease category and Nt indicates the number of species used in that category [40]. Its values vary between 0 and 1.

The use value of species is a quantitative method that shows the relative importance of locally known species. It is calculated according to the formula UV = Ui/N, where Ui is the number of reports of use mentioned by each informant (i) and N is the total number of surveyed informants for a given plant species [40].

## Results and discussion

### Diversity of medicinal plants

**Fig. 3:**
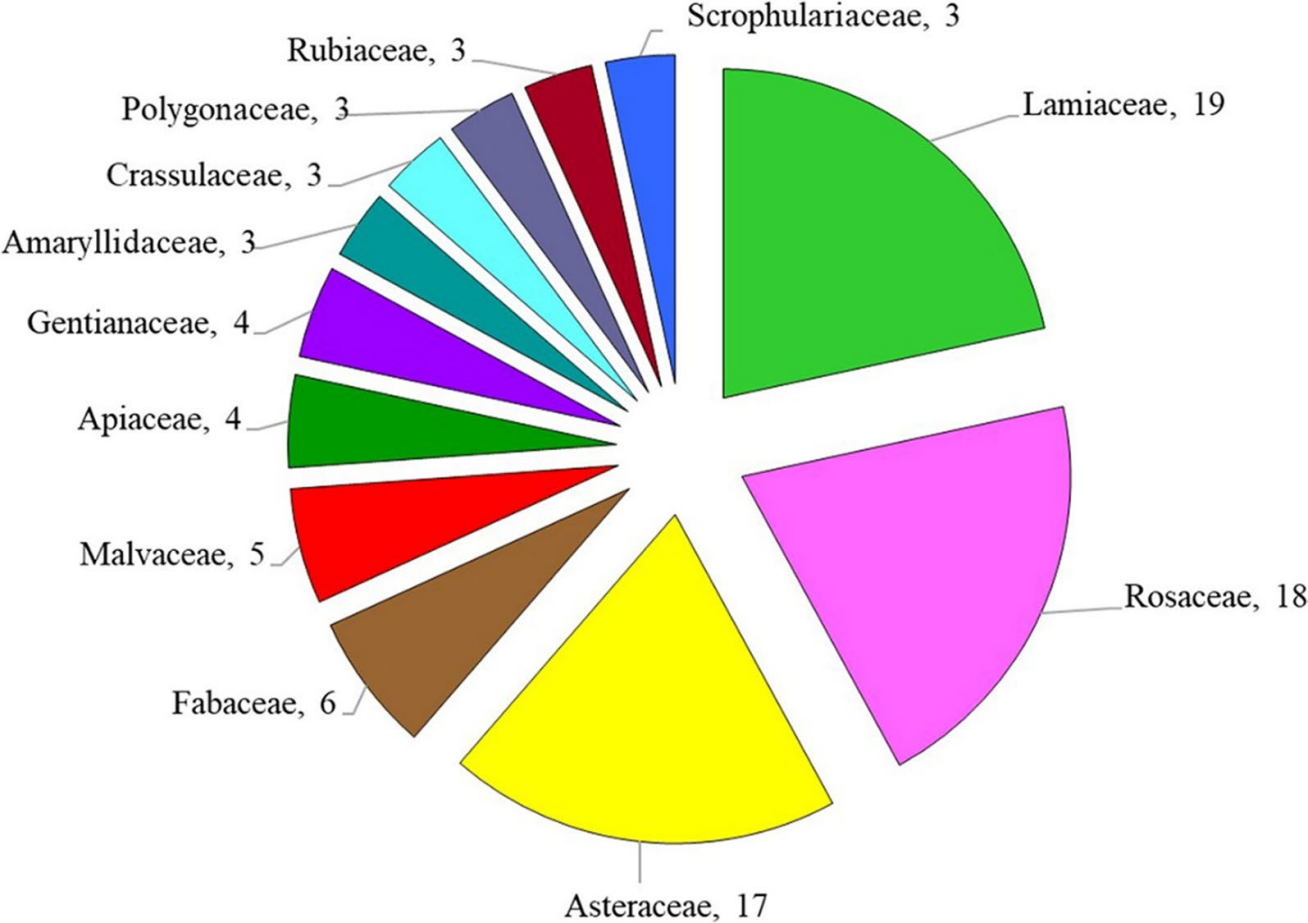
Dominant families of medicinal plants in the study area

Results of ethnobotanical research in the Mt Stara Planina region showed that 137 species classified into 51 families and 116 genera had practical applications in the ethnomedicine of the local population. Most species were Angiospermae (96.35%) and the total proportion of gymnosperms, pteridophytes, and lichen was 3.65% (Table 3). In terms of plant habit, 10 families, 17 genera, and 20 species were trees, 4 families, 6 genera, and 8 species were shrubs, 36 families, 92 genera, and 108 species were herbs, and 1 family, 1 genus, and 1 species was lichen (Tables 3 and 4). The richest families in terms of species were Lamiaceae (19), Rosaceae (18), and Asteraceae (17), which accounted for 39.42% of the total number of species. Twenty-nine families were represented by one species and 10 families by 2 species, while 12 families contained ≥ 3 plant species (Table 4, Fig. 3). The genera with the highest number of species were *Allium*, *Gentiana*, *Mentha*, and *Tilia* (three species each) (Table 4).

**Table 3:** Classification of medicinal plants in the study area

Category	Habit	Number of families	Number of genera	Number of species	Percentage of total species (%)
Angiospermae	Tree	9	15	18	13.14
	Shrub	3	5	7	5.11
	Herb	37	91	107	78.10
Gymnospermae	Tree	1	2	2	1.46
	Shrub	1	1	1	0.73
Pteridophyta	Herb	1	1	1	0.73
Lichens	Thalus	1	1	1	0.73
Total		53	116	137	100

**Table 4:** Traditional use of medicinal plants in the Mt Stara Planina region (south-eastern Serbia)

Botanical name and voucher specimen	Family	Local common name	Status, Habitus, IUCN	Part/product of plant	Form of preparation	Mode of application	Diseases treated/number of respondents (ICPC-2)	Number of informants	Use value index (UV)
*Achillea clypeolata* Sm St-pl-Ast-01/18BISS	Asteraceae	Žuta hajdučka trava, Raven	w/herb/E	Ap	Tea	O T	Pain respiratory system R01(1); Bleeding/haemorrhage NOS A10(2); Genital symptom/complt female oth.) X29(40); Trauma/injury NOS A80(2);	41	0.804
*Achillea millefolium* L St-pl-Ast-02/18IBISS	Asteraceae	Bela hajdučka trava	w/herb	Ap	Tea	O	Pain respiratory system R01(1); Asthma R96(1); Abdominal pain epigastric D02(1); Chronic enteritis/ulcerative colitis D94(1); Haematemesis/vomiting blood D14(1); Liver disease NOS D97(3); Urinary calculus U95(1); Cholecystitis/cholelithiasis D98(1); Flatulence/gas/belching D08(3); Dyspepsia/indigestion D07(1); Constipation D12(1); Haemorrhoids K96(1); Loss of appetite T03(1); Weakness/tiredness general A04(1); Menstruation irregular/frequent X07(10); Menopausal symptom/complaint X11(10);	29	0.568
*Agrimonia eupatoria* L St-pl-Ros-01/18BISS	Rosaceae	Petrovac, Ranjenik	w/herb	Ap	Tea	O	Jaundice D13(10); Liver disease NOS D97(2); Blood/lymph/spleen disease other B99(2); Stomach function disorder D87(1); Kidney symptom/complaint U14(1); Cholecystitis/cholelithiasis D98(2); Cystitis/urinary infection other U71(1); Diarrhoea D11(1); Infectious disease other/NOS A78(1); Health maintenance/prevention A98(1);	17	0.333
*Alchemilla vulgaris* L St-pl-Ros-02/18IBISS	Rosaceae	Virak	w/herb	Ap	Tea	O	Infertility/subfertility W15(5); Abortion spontaneous W82(1); Menstruation irregular/frequent X07(3); Menopausal symptom/complaint X11(6);	15	0.294
*Allium cepa* L St-pl-All-01/18IBISS	Amaryllidaceae	Crni luk	c/herb	Tu	Compress	T	Skin infection post-traumatic S11(11);	11	0.216
*Allium sativum* L St-pl-All-02/18IBISS	Amaryllidaceae	Beli luk	C/herb	Tu	Fresh	O	Health maintenance/prevention A98(26); Elevated blood pressure K85(34);	51	1
*Allium ursinum* L St-pl-All-03/18IBISS	Amaryllidaceae	Sremuš	w/herb/LC	Le	Fresh, tincture	O	Elevated blood pressure K85(34); Health maintenance/prevention A98(17);	51	1
*Althaea officinalis* L St-pl-Mal-01/18IBISS	Malvaceae	Beli slez	w/herb	Ro	Tea, decoction	O	Acute bronchitis/bronchiolitis R78(3); Laryngitis/tracheitis acute R77(2); Peptic ulcer other D86(1); Duodenal ulcer D85(1); Constipation D12(1); Health maintenance/prevention A98(1);	8	0.157
*Anacamptis morio* (L.) R.M.Bateman, Pridgeon & M.W.Chase St-pl-Orc-01/18IBISS	Orchidaceae	Kaćun, Salep	w/herb/NT	Tu	Tea	O	Diarrhoea D11(1);	1	0.019
*Arctium lappa* L St-pl-Ast-03IBISS	Asteraceae	Čičak veliki	w/herb	Ro	Tea	O	Liver disease NOS D97(1);	1	0.019
*Artemisia absinthium* L St-pl-Ast-04/18IBISS	Asteraceae	Beli pelin	w/herb	Ap	Tea Brandy—‘Pelinkovac’	O	Stomach function disorder D87(21); Liver disease NOS D97(5); Cholecystitis/cholelithiasis D98(5); Cystitis/urinary infection other U71(1); Menstruation irregular/frequent X07(1); Loss of appetite T03(4); Diabetes non-insulin dependent T90(1);	28	0.549
*Asarum europaeum* L St-pl-Ar-01/18IBISS	Aristolochiaceae	Kopitnjak	w/herb	Le	Tea, fresh leaves	O	Kidney symptom/complaint U14(1);	1	0.019
*Bellis perennis* L St-pl-Ast-05/18IBISS	Asteraceae	Bela rada	w/herb	Fl	Tea	T	Skin symptom/complaint other S29(2);	2	0.039
*Betula pubescens* Ehrh St-pl-Bet-01/18IBISS	Betulaceae	Bela breza	w/tree	Le	Tea	O	Joint symptom/complaint NOS L20(2); Urinary retention U08(1); Feeling anxious/nervous/tense P01(1); Weakness/tiredness general A04(1); Heart failure K77(1);	5	0.098
*Calendula officinalis* L St-pl-Ast-06/18IBISS	Asteraceae	Neven	c/herb	Fl	Tea Ointment	O T	For detoxification of the blood (tea) A98(1); Weakness/tiredness general A04(3); Varicose veins of leg K95(29);	33	0.647
*Capsella bursa-pastoris* Medik St-pl-Bra-01/18IBISS	Brassicaceae	Rusomača, Hoću-neću	w/herb	Ap	Tea, decoction	O	Bleeding/haemorrhage NOS A10(1); from the nose R06(3); Stomach function disorder D87(1); Intermenstrual bleeding X08(4); Haemorrhoids K96(2);	10	0.196
*Carlina acaulis* L St-pl-Ast-07/18IBISS	Asteraceae	Šeremetka, Vilino sito	w/herb	Ro	Fresh (it is eaten)	O	Cystitis/urinary infection other U71(1); Urinary calculus U95(1); Gout T92(1); Diabetes non-insulin dependent T90(6);	7	0.137
*Carlina vulgaris* L St-pl-Ast-08/18IBISS	Asteraceae	Obični kravljak	w/herb	Ap	Bath	T	Haemorrhoids K96(2);	2	0.039
*Centaurea cyanus* L St-pl-Ast-09/18IBISS	Asteraceae	Različak	w/herb	Fl	Tea	O T	Urinary retention U08(1); Conjunctivitis infectious F70(1); Eye infection/inflammation other F73(1);	2	0.039
*Centaurium erythraea* Rafn St-pl-Gen-01/18IBISS	Gentianaceae	Crveni kantarion, Kičica	w/herb	Ap	Tea Oil	O T	Loss of appetite T03(1); Dyspepsia/indigestion D07(1); Abdominal pain epigastric D02(2); Change faeces/bowel movements D18(2); Liver disease NOS D97(1); Splenomegaly B87(2); Flatulence/gas/belching D08(1); Diabetes non-insulin dependent T90(6); Diarrhoea D11(3); Health maintenance/prevention A98(5); Fever A03(5); Haemorrhoids K96(1); Limited function/disability (n) N28(1); Pain general/multiple sites A01(1) (applied externally as a liniment);	30	0.588
*Cetraria islandica* (L.) Ach St-pl-Par-01/18IBISS/lichen	Parmeliaceae	Islandski lišaj	w	WhPl	Tea, syrup	O	Cough R05(3);	3	0.059
*Chelidonium majus* L St-pl-Pap-01/18IBISS	Papaveraceae	Lišajevac, Rusa trava	w/herb	WhPl	Tea Juice (fresh)	O T	Rheumatoid/seropositive arthritis L88(1); Fibromyoma uterus X78(1); Kidney symptom/complaint U14(1); Liver disease NOS D97(2); Cholecystitis/cholelithiasis D98(2); Haemorrhoids K96(3); Malignant neoplasm stomach D74(4); Malignant neoplasm colon/rectum D75(4); Dysuria/painful urination U01(1); Tinnitus, ringing/buzzing ear H03(1); Health maintenance/prevention A98(1); Warts S03(24) (the juice is applied externally); Malignant neoplasm of skin S77(10); *Care should be taken when taken orally as *c. majus* is poisonous in larger doses. Most often only a small teacupful of the tea is drunk	34	0.666
*Cichorium intybus* L St-pl-Ast-10/18IBISS	Asteraceae	Vodopija	w/herb/LC	Ro	Tea	O	Loss of appetite T03(2); Diarrhoea D11(2); Liver disease NOS D97(3); Cholecystitis/cholelithiasis D98(3); Peptic ulcer other D86(2); Headache N01(1); Feeling anxious/nervous/tense P01(1); Heart failure K77(1); Health maintenance/prevention A98(9);	19	0.372
*Clinopodium nepeta* subsp. *spruneri* (Boiss.) Bartolucci & F.Conti St-pl-Lam-01/18IBISS	Lamiaceae	Divlji bosiljak, Verem trava	w/herb	Ap	Tea (one-component or tea blend: lavender, calamint, thyme and nettles)	O	Feeling anxious/nervous/tense P01(2);	2	0.039
*Cornus mas* L St-pl-Cor-01/18IBISS	Cornaceae	Dren	w/tree	Fr	Juice, pickling liquid	O	Iron deficiency anaemia B80(41);	41	0.804
*Corylus avellana* L St-pl-Bet-02/18IBISS	Betulaceae	Leska	w/tree	Le, Bk	Tea	O	Diarrhoea D11(5);	5	0.098
*Crataegus monogyna* Jacq St-pl-Ros-03/18IBISS	Rosaceae	Glog	w/tree	Fl, Le, Fr	Tea, juice	O	Heart failure K77 (38); Elevated blood pressure K85(3); Feeling anxious/nervous/tense P01(1);	38	0.745
*Cydonia oblonga* Mill St-pl-Ros-04/18IBISS	Rosaceae	Dunja	w/tree	Le	Tea	O	Diarrhoea D11(9);	9	0.176
*Delphinium consolida* L St-pl-Ran-03/18IBISS	Ranunculaceae	Žavornjak	w/herb	Ap	Tea	O	Prostatitis/seminal vesiculitis Y73(1);	1	0.019
*Dioscorea communis* (L.) Caddick & Wilkin St-pl-Dio-01/18IBISS	Dioscoreaceae	Bljušt	w/herb	Fr	Fresh	T	Gout T92(2);	2	0.039
*Elymus repens* (L.) Gould St-pl-Poa-01/18IBISS	Poaceae	Pirevina	w/herb	Ro	Tea	O	Pyelonephritis/pyelitis U70(1); Urethritis U72(1); Haemorrhoids K96(1); Benign prostatic hypertrophy Y85(1); Injury urinary tract U80(1);	4	0.078
*Epilobium angustifolium* L St-pl-Ona-01/18IBISS	Onagraceae	Kiprovina	w/herb	Ap	Tea	O	Benign prostatic hypertrophy Y85(5);	5	0.098
*Epilobium parviflorum* Schreb St-pl-Ona-02/18IBISS	Onagraceae	Svilovina, Vrbovka	w/herb	Ap	Tea	O	Prostate symptom/complaint Y06(3); Bladder symptom/complaint other U13(3); Kidney symptom/complaint U14(3);	6	0.117
*Equisetum arvense* L St-pl-Equ-01/18IBISS	Equisetaceae	Rastavić	w/herb/LC	Ap	Tea	O	Urinary retention U08(9);	9	0.176
*Euphrasia officinalis* L St-pl-Oro-01/18IBISS	Orobanchaceae	Vidac	w/herb	Ap	Tea	T	Eye infection/inflammation other F73(1);	1	0.019
*Filipendula ulmaria* (L.) Maxim St-pl-Ros-05/18IBISS	Rosaceae	Medunika	w/herb	Fl	Tea	O	Fibromyoma uterus X78(1); Heartburn D03(9); Peptic ulcer other D86(3);	12	0.235
*Foeniculum vulgare* Mill St-pl-Api-02/18IBISS	Apiaceae	Komorač	w/herb	Se	Tea; seeds are added to food	O	Flatulence/gas/belching D08(4);	4	0.078
*Fragaria vesca* L St-pl-Ros-07/18IBISS	Rosaceae	Divlja jagoda	w/herb/LC	Le, Fr	Tea; the fruits are eaten raw	O	Diarrhoea D11(1); Gastrointestinal infection D70(1); Urinary retention U08(3); Haemorrhoids K96(1); Health maintenance/prevention A98(1);	8	0.157
*Frangula alnus* Mill St-pl-Rh-01/18IBISS	Rhamnaceae	Krušina	w/tree	Bk (dried and left to stand for at least a year)	Tea	O	Change faeces/bowel movements D18(2);	2	0.039
*Fumaria officinalis* L St-pl-Fu-01/18IBISS	Papaveraceae	Dimnjača	w/herb	Ap	Tea (used for only 7–8 days)	O	Health maintenance/prevention A98(1); Liver disease NOS D97(2);	3	0.059
*Galega officinalis* L St-pl-Fab-02/18IBISS	Fabaceae	Ždraljevina	w/herb	Ap	Tea	O	Diabetes non-insulin dependent T90(2);	2	0.039
*Galium aparine* L St-pl-Rub-02/18IBISS	Rubiaceae	Broć, lepljivac	w/herb	Ap	Tea	T	Trauma/injury NOS A80(3); Lymph gland(s) enlarged/painful B02(1); Malignant neoplasm of skin S77 (1); Neoplasm skin benign/unspecified S79(1); Acne S96(1); Boil/carbuncle S10(2); Corn/callosity S20(1);	7	0.137
*Galium odoratum* Scop St-pl-Rub-1/18IBISS	Rubiaceae	Lazarkinja	w/herb	Ap	Tea	O	Headache N01(1); Sleep disturbance P06(1);	1	0.019
*Galium verum* L St-pl-Rub-03IBISS	Rubiaceae	Ivanjsko cveće	w/herb	Ap	Tea	O T	Hypothyroidism/myxoedema T86(2); Malig. neoplasm digest other/NOS D77(1); Kidney symptom/complaint U14(1); Liver disease NOS D97(1); Blood/lymph/spleen disease other B99(1); Leukaemia B73(1); Epilepsy N88(1); Stomach function disorder D87(1); Feeling anxious/nervous/tense P01(1); Skin injury other S19(8); Burn/scald S14(18); Boil/carbuncle S10(7) Malignant neoplasm of skin S77(2);	39	0.765
*Gentiana asclepiadea* L St-pl-Gen-01/18IBISS	Gentianaceae	Plava lincura, Trava od žutice, Svećica	w/herb	Ap, Ro	Herb brandy ‘Travarica’ (1 small glassful is drunk in the morning); Medicinal mixture: a kilogram of honey is mixed with approx. 50 g of the powdered root One teaspoonful is taken (sucked) three times a day, under the tongue, before food. Instead of honey, the powdered root can be drunk with a little water, before food	O	Stomach function disorder D87(1); Malignancy NOS A79(51); Liver disease NOS D97(5); Abdominal pain/cramps general D01(5); Jaundice D13(7); Health maintenance/prevention A98(2);	51	1
*Gentiana cruciata* L St-pl-Gen-02/18IBISS	Gentianaceae	Otodovka	w/herb	Ap	Herb brandy ‘Travarica’; Tea;	O	Stomach function disorder D87(6); Malignancy NOS A79(51); *A panacea* – Feeling anxious/nervous/tense P01(4); Diabetes non-insulin dependent T90(3); Health maintenance/prevention A98(2); Worms/other parasites D96(5); Genital pain female X01(1); Liver disease NOS D97(4); Cholecystitis/cholelithiasis D98(4); Prostate symptom/complaint Y06(4); Change faeces/bowel movements D18(1); Cough R05(1); Loss of appetite T03(2);	51	1
*Gentiana lutea* L St-pl-Gen-03/18IBISS	Gentianaceae	Lincura	w/herb/LC/HD	Ro	‘Gentian brandy’ (approx 50–100 g of root should be left to sit for 3 weeks in 1 L of twice-distilled brandy; one small glassful is drunk three times a day)	O	Dyspepsia/indigestion D07(34); Stomach function disorder D87(17);	51	1
*Geranium robertianum* L St-pl-Ger-01/18IBISS	Geraniaceae	Crveni zdravac	w/herb	Ap	Tea	O	Injury urinary tract U80(3); Teeth/gum symptom/complaint D19(1); Mouth/tongue/lip symptom/complt. D20(1); Jaundice D13(2); Swollen ankles/oedema K07(1); Health maintenance/prevention A98(1);	7	0.137
*Geum urbanum* L St-pl-Ros-08/18IBISS	Rosaceae	Zečija stopa	w/herb	Ro, Ap	Tea	O	Health maintenance/prevention A98(1);	1	0.019
*Glechoma hederacea* L St-pl-Lam-02/18IBISS	Lamiaceae	Dobričica	w/herb	Ap	Tea	O	Respiratory symptom/complaint oth. R29(2);	2	0.039
*Hedera helix* L St-pl-Ara-01/18IBISS	Araliaceae	Bršljan	w/herb	Le	Decoction	T	Allergy/allergic reaction NOS A92(1);	1	0.019
*Heracleum sphondylium* L St-pl-Api-03/18IBISS	Apiaceae	Mečija šapa	w/herb	Ro	Tea	O	Epilepsy N88(1);	1	0.019
*Herniaria glabra* L St-pl-Car-01/18IBISS	Caryophyllaceae	Kilavica, Sitnica	w/herb	WhPl	Tea	O	Urinary retention U08(1);	1	0.019
*Humulus lupulus* L St-pl-Can-01/18IBISS	Cannabaceae	Hmelj	w/herb	Le, Fl	Tea	O	Feeling anxious/nervous/tense P01(2);	2	0.039
*Hylotelephium spectabile* (Boreau) H.Ohba St-pl-Cras-01/18IBISS	Crassulaceae	Debela koka	w/c/herb	Le	Compress (the epidermis is removed from the leaf)	T	Boil/carbuncle S10(15); Skin injury other S19(17); Laceration/cut S18(3);	29	0.568
*Hypericum perforatum* L St-pl-Hyp-01/18IBISS	Hypericaceae	Kantarion	w/herb	Ap	Tea, Oil	O T O/T	Peptic ulcer other D86(1); Liver disease NOS D97(2); Cholecystitis/cholelithiasis D98(2); Speech disorder N19(2); Sleep disturbance P06(4); Feeling anxious/nervous/tense P01(1); Bedwetting/enuresis P12(1); Burn/scald S14(42) (applied externally); Laceration/cut S18(2); Skin injury other S19(35); Ear pain/earache H01(2) (the oil is used in the form of drops to treat earache); Haemorrhoids K96(2); Pain respiratory system R01(1); Headache N01(1); Health maintenance/prevention A98(4);	51	1
*Hyssopus officinalis* L St-pl-Lam-03/18IBISS	Lamiaceae	Miloduh, Izop	w/c//herb	Ap	Tea	O	Chest pain NOS A11(2); Pain respiratory system R01(4);	4	0.078
*Inula helenium* L St-pl-Ast-12/18IBISS	Asteraceae	Beli oman	w/herb	Le, Fl	Tea Tincture	O T	Pain respiratory system R01(1); Sputum/phlegm abnormal R25(1); Health maintenance/prevention A98(1) (tea); Rheumatoid/seropositive arthritis rheumatism L88(4); Limited function/disability (n) N28(1);	6	0.117
*Juglans regia* L St-pl-Jug-01/18IBISS	Juglandaceae	Orah	w/c/tree	Le, Fr	Tea; the fruits are eaten raw; Walnut brandy – ‘Orahovača’	O	Health maintenance/prevention A98(2);	2	0.039
*Juniperus communis* L St-pl-Cup-01/18IBISS	Cupressaceae	Kleka	w/shrub	Fr Ro	Juniper brandy – ‘Klekovača’; Tea Balm	O T	‘Klekovača’: Health maintenance/prevention A98(13) and for massaging (improves circulation) – taken before breakfast; Gastrointestinal infection D70(3); Tea: Prostatitis/seminal vesiculitis Y73(3); Urinary retention U08(2); Cystitis/urinary infection other U71(1); Cholecystitis/cholelithiasis D98(D) 1; Chronic bronchitis R79(3); Rheumatoid/seropositive arthritis L88(7);	17	0.333
*Leonurus cardiaca* L St-pl-Lam-04/18IBISS	Lamiaceae	Srdačica	w/herb	Ap	Tea	O T (a compress)	Paroxysmal tachycardia K79(4); Cardiac arrhythmia NOS K80(4); Feeling anxious/nervous/tense P01(2); Burn/scald S14(2); Skin injury other S19(2); Boil/carbuncle S10(2); Dermatitis/atopic eczema S87(2);	6	0.117
*Linum* sp. St-pl-Lin-01/18IBISS	Linaceae	Lan	w/herb	Se	Tea	O	Constipation D12(2); Weight loss T08(1);	3	0.059
*Lycopodium clavatum* L St-pl-Lyc-01/18IBISS	Lycopodiaceae	Prečica, Crvotočina	w/herb/LC	WhPl	Tea (only one cup drunk a day, before breakfast)	O	Pain general/multiple sites A01(1); Liver disease NOS D97(1); Chronic ulcer skin S97(1);	1	0.019
*Lycopus europaeus* L St-pl-Lam-05/18IBISS	Lamiaceae	Gagamija	w/herb/LC	Ap	Tea	O	Hyperthyroidism/thyrotoxicosis T85(1);	1	0.019
*Lythrum salicaria* L St-pl-Lyt-01/18IBISS	Lythraceae	Vrbica, Tvrdac	w/herb/LC	Ap	Tea	O	Diarrhoea D11(1); Abdominal pain epigastric D02(2); Chronic enteritis/ulcerative colitis D94(2); Feeling anxious/nervous/tense P01(1); Genital pain female X01(1); Intermenstrual bleeding X08(2); Sexual function sympt./complt.(m) Y08(1); Cystitis/urinary infection other U71(1);	6	0.117
*Malus sylvestris* (L.) Mill St-pl-Ros-09/18IBISS	Rosaceae	Divlja jabuka	w/tree/DD	Fr Le	Pickling juice; Vinegar Compress	O T	Health maintenance/prevention A98(2); Trauma/injury NOS A80(2);	3	0.059
*Malva sylvestris* L St-pl-Mal-02/18IBISS	Malvaceae	Crni slez	w/herb	Fl Le	Decoction (for cleaning and as a compress); Cataplasm (a mash is made from mallow leaves and barley flour)	T	Skin injury other S19(3); Benign neoplasm thyroid T72(1);	4	0.078
*Marrubium vulgare* L St-pl-Lam-06/18IBISS	Lamiaceae	Marulja, Očajnica	w/herb	Ap	Tea	O	Infertility/subfertility W15(2);	2	0.039
*Matricaria chamomilla* L St-pl-Ast-13/18IBISS	Asteraceae	Kamilica	w/herb	Ap	Tea	O	Pain respiratory system R01 (5); Influenza R80 (12);	15	0.294
*Melilotus albus* Medik St-pl-Fab-04/18IBISS	Fabaceae	Beli kokotac	w/herb/LC	Ap	Tea	O	Heart pain K01(1); Varicose veins of leg K95(1);	1	0.019
*Melilotus officinalis* (L.) Pall St-pl-Fab-03/18IBISS	Fabaceae	Žuti kokotac	w/herb/LC	Ap	Tea	O	Heart pain K01(1); Varicose veins of leg K95(5);	5	0.098
*Melissa officinalis* L St-pl-Lam-07/18IBISS	Lamiaceae	Matičnjak, Matočina	w/herb	Ap	Tea	O	Pain respiratory system R01(22); Feeling anxious/nervous/tense P01(5);	27	0.529
*Mentha* × *piperita* L St-pl-Lam-08/18IBISS	Lamiaceae	Pitoma nana	w/c/herb	Ap	Tea	O	Stomach function disorder D87(40); Pain respiratory system R01(4); Cough R05(7); Acute bronchitis/bronchiolitis R78(3); Chronic bronchitis R79(3); Influenza R80(22);	50	0.980
*Mentha pulegium* L St-pl-Lam-09/18IBISS	Lamiaceae	Metvica	w/herb/LC	Ap	Tea	O	Stomach function disorder D87(12);	12	0.235
*Mentha spicata* L St-pl-Lam-10/18IBISS	Lamiaceae	Divlja nana	w/herb/LC	Ap	Tea	O	Pain respiratory system R01(2); Acute bronchitis/bronchiolitis R78(2); Chronic bronchitis R79(2); Cough R05(1); Stomach function disorder D87(3);	8	0.157
*Morus nigra* L St-pl-Mor-01/18IBISS	Moraceae	Crni dud	w/c/tree	Le	Tea	O	Diabetes non-insulin dependent T90(17);	17	0.333
*Nasturtium officinale* R.Br St-pl-Bra-02/18IBISS	Brassicaceae	Potočarka	w/herb	Le	Juice	T	Acne S96(1);	1	0.019
*Ocimum basilicum* L St-pl-Lam-12/18IBISS	Lamiaceae	Bosiljak	c/herb	Ap	Tea	O	Feeling anxious/nervous/tense P01(2); Sneezing/nasal congestion R07(9);	11	0.216
*Ononis spinosa* L St-pl-Fab-05/18IBISS	Fabaceae	Zečiji trn	w/shrub	Ap	Tea	O	Urinary retention U08(2); Cystitis/urinary infection other U71(2); Urinary calculus U95(1);	3	0.059
*Origanum vulgare* L St-pl-Lam-13/18IBISS	Lamiaceae	Vranilovka	w/herb	Ap	Tea, decoction	O	Influenza R80(3); Pain respiratory system R01(2); Strep throat R72(4); Cough R05(3); Acute bronchitis/bronchiolitis R78(3); Chronic bronchitis R79(3); Abdominal pain/cramps general D01(2); Dyspepsia/indigestion D07(2); Jaundice D13(2); Anxiety disorder/anxiety state P74(1); Tinnitus, ringing/buzzing ear H03(1); Headache N01(1); Infertility/subfertility male Y10(1);	21	0.412
*Oxalis acetosella* L St-pl-Oxa-01/18IBISS	Oxalidaceae	Zečija soca	w/herb	Le (only fresh)	Tea, juice	O	Jaundice D13(1); Pyelonephritis/pyelitis U70(1); Dermatitis/atopic eczema S87(1);	3	0.059
*Persicaria bistorta* Samp St-pl-Polygo-02/18IBISS	Polygonaceae	Srčanik, Srčenica	w/herb	Rh	Tea	O	Bleeding/haemorrhage NOS A10(2); Malignancy NOS A79(2); Health maintenance/prevention A98(2); Diarrhoea D11(3); Stomach function disorder D87(3); Heart pain K01(1); Genital pain female X01(16); Fibromyoma uterus X78(5);	26	0.510
*Peucedanum* sp. St-pl-Api-05/18IBISS	Apiaceae	Raskovnik	w/herb	Ap	Tea, decoction	O	Mouth/tongue/lip symptom/complt. D20(1); Haematemesis/vomiting blood D14(1); Haemorrhoids K96(1); Nosebleeds/epistaxis R06(1); Urinary calculus U95(1); Urinary frequency/urgency U02(1); Menstruation excessive X06(1); Infertility/subfertility male Y10(1);	6	0.117
*Phaseolus vulgaris* L St-pl-Fab-06/18IBISS	Fabaceae	Pasulj	c/herb	Fr (empty pods)	Tea	O	Diabetes non-insulin dependent T90(1);	1	0.019
*Picea abies* (L.) H.Karst St-pl-Pin-01/18IBISS	Pinaceae	Smrča	w/tree	Re	Compress	T	Skin texture symptom/complaint S21(4); Trauma/injury NOS A80(2);	4	0.078
*Pinus nigra* J.F.Arnold St-pl-Pin-02/18 IBISS	Pinaceae	Crni bor	w/tree	Ne, Re	Syrup (needles) Compress (resin)	O T	Cough R05(4) (syrup); Skin texture symptom/complaint S21(5) (resin);	5	0.098
*Plantago lanceolata* L St-pl-Pla-01/18IBISS	Plantaginaceae	Ženska bokvica	w/herb	Le	Tea, syrup Mixed with honey Compress	O T	Cough R05(5); Urinary frequency/urgency U02(2); Health maintenance/prevention A98(4); Diarrhoea D11(2); Chronic enteritis/ulcerative colitis D94(2); Abdominal pain/cramps general D01(1); Duodenal ulcer D85(3); Genital pain female X01(1); Abdominal pain/cramps general D01(1) (the leaves are ground and mixed with honey); Trauma/injury NOS A80(31); Skin infection other S76(41); Chronic ulcer skin S97(31);	49	0.961
*Plantago major* L St-pl-Pla-02/18IBISS	Plantaginaceae	Muška bokvica	w/herb	Le	Tea, syrup Mixed with honey Compress	O T	Cough R05(5); Urinary frequency/urgency U02(2); Health maintenance/prevention A98(4); Diarrhoea D11(2); Chronic enteritis/ulcerative colitis D94(2); Abdominal pain/cramps general D01(1); Duodenal ulcer D85(3); Genital pain female X01(1); Abdominal pain/cramps general D01(1) (the leaves are ground and mixed with honey); Trauma/injury NOS A80(31); Skin infection other S76(41); Chronic ulcer skin S97(31);	49	0.961
*Polygonum aviculare* L St-pl-Polygo-01/18IBISS	Polygonaceae	Troskot	w/herb	Ap	Tea	O	Health maintenance/prevention A98(1); Diarrhoea D11(1); Rectal bleeding D16(1); Jaundice D13(1); Rheumatoid/seropositive arthritis L88(1); Chronic obstructive pulmonary dis R95(1); Urinary calculus U95(1); Kidney symptom/complaint U14(1); Gout T92(1);	5	0.098
*Polypodium vulgare* L St-pl-Polyp-01/18IBISS	Polypodiaceae	Slatka paprat	w/herb	Rh	Tea	O	Acute bronchitis/bronchiolitis R78(1); Chronic obstructive pulmonary dis R95(1);	1	0.019
*Potentilla erecta* (L.) Raeusch St-pl-Ros-10/18IBISS	Rosaceae	Trava steža	w/herb	Rh	Decoction	O T	Abdominal pain/cramps general D01(1); Bleeding/haemorrhage NOS A10(2); Diarrhoea D11(1); Mouth/tongue/lip symptom/complt. D20(1); Haemorrhoids K96(2); Skin injury other S19(3);	9	0.176
*Primula veris* L St-pl-Pri-02/18IBISS	Primulaceae	Jagorčevina	w/herb	Ro, Fl	Tea, syrup	O	Acute bronchitis/bronchiolitis R78(13); Cough R05(6); Feeling anxious/nervous/tense P01(1);	13	0.255
*Prunus avium* (L.) L St-pl-Ros-11/18IBISS	Rosaceae	Trešnja	w/c/tree/LC	Fr	Herb brandy ‘Travarica’	O	Dyspepsia/indigestion D07(1); Health maintenance/prevention A98(1);	1	0.019
*Prunus spinosa* L St-pl-Ros-12/18IBISS	Rosaceae	Trnjina	w/shrub/LC	Fr	Juice, tea, the fruits are eaten raw	O	Headache N01(1); Heart failure K77(3); Cholecystitis/cholelithiasis D98(4); Diabetes non-insulin dependent T90(4);	10	0.196
*Pulmonaria officinalis* L St-pl-Bor-02/18IBISS	Boraginaceae	Plućnjak	w/herb	Ap	Tea	O	Asthma R96(14); Acute bronchitis/bronchiolitis R78(8); Tuberculosis A70(3); Cough R05(2);	21	0.412
*Pyrus pyraster* (L.) Burgsd St-pl-Ros-13/18IBISS	Rosaceae	Divlja kruška	w/tree/LC	Le, Bk Fr	Tea Pickling juice ‘vinegar’; Herb brandy ‘Travarica’	O	Lipid disorder T93(31); Diabetes non-insulin dependent T90(12); Stomach function disorder D87(1); Health maintenance/prevention A98(3);	33	0.647
*Rosa canina* L St-pl-Ros-14/18IBISS	Rosaceae	Šipak, Divlja ruža	w/shrub	Fr	Tea	O	Pain respiratory system R01(16); Influenza R80(12);	16	0.314
*Rubus idaeus* L St-pl-Ros-16/18IBISS	Rosaceae	Malina	w/shrub	Le, Fr	Tea, the fruits are eaten raw	O	Fever A03(1); Laryngitis/tracheitis acute R77(1); Diarrhoea D11(1); Rectal bleeding D16(1); Menstruation excessive X06(3); Uncomplicate labour/delivery still W91(1);	7	0.137
*Rubus plicatus* Weihe & Nees St-pl-Ros-15/18IBISS	Rosaceae	Kupina	w/shrub	Le, Fr	Tea; the fruits are eaten raw	O	Health maintenance/prevention A98(7); Iron deficiency anaemia B80(13); Pain respiratory system R01(1); Cough R05(1); Laryngitis/tracheitis acute R77(1); Dyspepsia/indigestion D07(3); Diarrhoea D11(2); Mouth/tongue/lip symptom/complt. D20(1); Genital pain female X01(1); Menstruation irregular/frequent X07(3); Elevated blood pressure K85(2);	18	0.353
*Rumex patientia* L St-pl-Polygo-03/18IBISS	Polygonaceae	Zelje	w/herb	Se	Tea	O	Diarrhoea D11(9);	9	0.176
*Ruta graveolens* L St-pl-Rut-01/18 IBISS	Rutaceae	Ruta	c/herb	Le	Only two leaves are eaten fresh during the day	O	Diarrhoea D11(1);	1	0.019
*Salix alba* L St-pl-Sal-01/18IBISS	Salicaceae	Bela vrba	w/tree	Br (with young shoots) – ‘willow charcoal’ Le, Bk	Tea	O	Diarrhoea D11(1); Cough R05(1);	1	0.019
*Salvia officinalis* L St-pl-Lam-14/18IBISS	Lamiaceae	Žalfija	c/herb	Ap	Tea	O T	Abdominal pain/cramps general D01(21); Menstrual pain X02(5); Fever A03(2); Tuberculosis A70(3); Liver disease NOS D97(2); Kidney symptom/complaint U14(2); Infectious disease other/NOS A78(28);	30	0.588
*Salvia verticillata* L St-pl-Lam-15/18IBISS	Lamiaceae	Pršljenasta kadulja, Seruša	w/herb	Le	Compress	T	Trauma/injury NOS A80(2);	2	0.039
*Sambucus ebulus* L St-pl-Ado-01/18IBISS	Viburnaceae	Burjan	w/herb	Fr	Conserve, jam (must be measured out, 1 teaspoon, in the morning);	O	Malignancy NOS A79(8);	8	0.157
*Sambucus nigra* L St-pl-Ado-02/18IBISS	Viburnaceae	Zova	w/tree	Fl	Tea	O	Pain respiratory system R01(4); Cough R05(5); Strep throat R72(4); Rheumatoid/seropositive arthritis L88(1); Urinary retention U08(1);	13	0.255
*Sanguisorba minor* Scop St-pl-Ros-17/18IBISS	Rosaceae	Dinjica, Lubeničarka	w/herb	WhPl	Tea	T	Animal/human bite S13(4);	4	0.078
*Sanicula europaea* L St-pl-Api-06/18IBISS	Apiaceae	Milogled	w/herb	Ro, WhPl	Tea Tincture	O T (compress)	Health maintenance/prevention A98(1); Bleeding/haemorrhage NOS A10(1); Strep throat R72(1); Trauma/injury NOS A80(2); Skin texture symptom/complaint S21(1); Gout T92(1);	4	0.078
*Satureja montana* L St-pl-Lam-16/18IBISS	Lamiaceae	Čubrika, Rtanjski čaj	w/herb	Ap	Tea	O	Cough R05(35); Sputum/phlegm abnormal R25(2); Dyspepsia/indigestion D07(3); Flatulence/gas/belching D08(3); Anxiety disorder/anxiety state P74(4);	45	0.882
*Scrophularia nodosa* L St-pl-Scr-01/18IBISS	Scrophulariaceae	Stupnik, Crna kopriva	w/herb	Le	Tea (just for gargling)	O	Goitre T81(1);	1	0.019
*Sedum acre* L St-pl-Cras-02/18IBISS	Crassulaceae	Žednjak	w/herb	Ap	Tea	T	Haemorrhoids K96(1);	1	0.019
*Sempervivum tectorum* L St-pl-Cras-03/18IBISS	Crassulaceae	Čuvarkuća	w/c/herb	Le	Juice	T	Otitis externa H70(6); Acute otitis media/myringitis H71(6); Ear pain/earache H01(6); for skin complaints S99(28); Haemorrhoids K96(16);	50	0.980
*Solidago virgaurea* L St-pl-Ast-15/18IBISS	Asteraceae	Šumska zlatnica, Čelebi grana	w/herb	Ap	Tea	O	Cholecystitis/cholelithiasis D98(2); Cystitis/urinary infection other U71(1);	3	0.059
*Sorbus domestica* L St-pl-Ros-18/18IBISS	Rosaceae	Oskoruša, Jarebika	w/tree	Fr (the fruits should to overripen)	The fruits are eaten raw	O	Chronic enteritis/ulcerative colitis D94(4); Constipation D12(6);	8	0.157
*Sorbus torminalis* (L.) Crantz St-pl-Ros-19/18IBISS	Rosaceae	Brekinja	w/tree	Bk	Tea	O	Diabetes non-insulin dependent T90(5);	5	0.098
*Stachys officinalis* (L.) Trevis. St-pl-Lam-17/18IBISS	Lamiaceae	Ranilist, Ranjenik	w/herb	Le	Tea Compress	O T	Health maintenance/prevention A98(1); Jaundice D13(1); Asthma R96(1); Epilepsy N88(1); Headache N01(1); Heartburn D03(1); Malignancy NOS A79(1); Rheumatoid/seropositive arthritis L88(1); Trauma/injury NOS A80(14);	18	0.353
*Symphytum officinale* L St-pl-Bor-03/18IBISS	Boraginaceae	Crni gavez	w/herb	Ro	Ointment, compress (the ground root is cooked in milk and left to stand overnight; the dressing is changed every second day over a period of two months)	T	Joint symptom/complaint NOS L20(9); Bone fractures: Fracture: radius/ulna L72(15); Fracture: tibia/fibula L73(15); Fracture: hand/foot bone L74(15); Fracture: femur L75(15); Fracture: other L76(15);	20	0.392
*Tanacetum vulgare* L St-pl-Ast-17/18IBISS	Asteraceae	Povratić	w/herb	Ap	Tea	O	Abortion induced W83(1);	1	0.019
*Taraxacum officinale* F.H.Wigg St-pl-Ast-18/18IBISS	Asteraceae	Maslačak	w/herb	Ro Le, Fl	Tea Conserve, honey, salad	O	Constipation D12(8); Urinary retention U08(4); Cholecystitis/cholelithiasis D98(12); Lipid disorder T93(31); Health maintenance/prevention A98(5);	48	0.941
*Telekia speciosa* (Schreb.) Baumg St-pl-Ast-19/18IBISS	Asteraceae	Oman	w/herb	Ro	Tea, tincture	O	Acute bronchitis/bronchiolitis R78(6); Asthma R96(11);	13	0.255
*Teucrium chamaedrys* L St-pl-Lam-18/18IBISS	Lamiaceae	Podubica	w/herb	Ap	Tea	O	Stomach function disorder D87(14); Acute bronchitis/bronchiolitis R78(6); Health maintenance/prevention A98(1);	21	0.412
*Teucrium montanum* L St-pl-Lam-19/18IBISS	Lamiaceae	Trava iva	w/herb	Ap	Tea	O	Health maintenance/prevention A98(2); Cholecystitis/cholelithiasis D98(5); Haemorrhoids K96(6); Cough R05(12); Gastrointestinal infection D70(13); Diabetes non-insulin dependent T90(7);	34	0.666
*Thymus serpyllum* L St-pl-Lam-20/18IBISS	Lamiaceae	Majčina dušica, Babina dušica	w/herb	Ap	Tea	O	Pain respiratory system R01(51); Influenza R80(12); Feeling anxious/nervous/tense P01(5); Infectious disease other/NOS A78(1); Heartburn D03(3); Menstruation irregular/frequent X07(5); Skin symptom/complaint other S29(3);	51	1
*Tilia cordata* Mill St-p1-Til-01/18IBISS	Malvaceae	Sitnolisna lipa	w/tree	Le, Fl	Tea	O	Pain respiratory system R01(33); Cough R05(6);	39	0.765
*Tilia platyphyllos* Scop St-p1-Til-02/18IBISS	Malvaceae	Krupnolisna lipa	w/tree	Le, Fl	Tea	O	Pain respiratory system R01(12); Cough R05(6);	18	0.353
*Tilia tomentosa* Moench St-p1-Til-03/18IBISS	Malvaceae	Srebrna lipa	w/tree	Le, Fl	Tea	O	Pain respiratory system R01(12); Cough R05(6);	18	0.353
*Trifolium pratense* L St-pl-Fab-07/18IBISS	Fabaceae	Crvena detelina	w/herb	Fl	Tea	O	Asthma R96(1); Cough R05(1); Health maintenance/prevention A98(1); Malignancy NOS A79(1); Feeling anxious/nervous/tense P01(1); Genital pain female X01(2);	7	0.137
*Tussilago farfara* L St-pl-Ast-20/18IBISS	Asteraceae	Podbel	w/herb	Le	Tea Compress	O T	Cough R05(15); Chronic obstructive pulmonary dis R95(3); Haemorrhoids K96(2) 2;	17	0.333
*Urtica dioica* L St-pl-Urt-01/18IBISS	Urticaceae	Kopriva	w/herb	Ap	Tea, medicinal mixture (the tips of young shoots are mixed with approx. 15 walnuts: the mixture is ground thoroughly and then eaten)	O	Rheumatoid/seropositive arthritis L88(1); Leukaemia B73(12); Iron deficiency anaemia B80(40); Allergy/allergic reaction NOS A92(1); Health maintenance/prevention A98(11); Goitre T81(1); Jaundice D13(6); Varicose veins of leg K95(4); Weakness/tiredness general A04(5); Lipid disorder T93(3); Elevated blood pressure K85(3); Breast/lactation symptom/complaint W19(2); Haemorrhoids K96(5); Peptic ulcer other D86(1); Ischaemic heart disease w. angina K74(1); Diarrhoea D11(4); Diabetes non-insulin dependent T90(2); Health maintenance/prevention A98(1);	51	1
*Vaccinium myrtillus* L St-pl-Eri-01/18IBISS	Ericaceae	Borovnica	w/shrub	Le Fr	Tea Conserve, juice, or eaten as fresh or dried fruit	O	Urethritis U72(2); Urinary calculus U95(1); Urinary retention U08(1); Health maintenance/prevention A98(46);	48	0.941
*Vaccinium vitis-idaea* L St-pl-Eri-02/18IBISS	Ericaceae	Brusnica	w/shrub	Le, Fr Fr	Tea, decoction Pickling juice; the fruits are eaten raw	O	Infectious disease other/NOS A78(13); Kidney symptom/complaint U14(2); Bladder symptom/complaint other U13(2); Urethritis U72(5); Chronic enteritis/ulcerative colitis D94(2); to strengthen the heart muscle K77(3); Diabetes non-insulin dependent T90(6); Urinary calculus U95(3); Health maintenance/prevention A98(4);	36	0.706
*Valeriana officinalis* L St-pl-Val-01/18IBISS	Caprifoliaceae	Odoljen, Macina trava	w/herb	Ro	Tea	O	Feeling anxious/nervous/tense P01(11);	11	0.216
*Verbascum densiflorum* Bertol St-pl-Scr-02/18IBISS	Scrophulariaceae	Divizma	w/herb	Fl	Tea	O	Cough R05(2); Acute bronchitis/bronchiolitis R78(2); Pain respiratory system R01(2); Chronic enteritis/ulcerative colitis D94(1); Urinary retention U08(1);	6	0.117
*Verbascum phlomoides* L St-pl-Scr-03/18IBISS	Scrophulariaceae	Divizma, Svećnjak	w/herb	Fl	Tea	O	Cough R05(2); Acute bronchitis/bronchiolitis R78(2); Pain respiratory system R01(2); Chronic enteritis/ulcerative colitis D94(1); Urinary retention U08(1);	4	0.078
*Verbena officinalis* L St-pl-Ver-01/18IBISS	Verbenaceae	Verbena	w/herb	Ap	Tea	O	Feeling anxious/nervous/tense P01(1); Rheumatoid/seropositive arthritis L88(1); Urinary retention U08(1);	3	0.059
*Viola tricolour* L St-pl-Vio-02/18IBISS	Violaceae	Dan i noć, Žuta ljubičica	w/herb	Ap	Tea (for a rinse and as a compress)	T	Dermatitis/atopic eczema S87(2); Skin infection other S76(2);	3	0.059
*Zea mays* L St-pl-Po-02/18IBISS	Poaceae	Kukuruz	c/herb	Fl (♀, Corn silk)	Tea	O	Urinary calculus U95(5); Urinary retention U08(5);	5	0.098

*c* Cultivated species, *ws* Wild species, *Ap* Aerial part, *Bk* Bark, *Br* Branches, *Fl* Flower, *Fr* Fruit, *Le* Leaf, *Ne* Needles, *Re* Resin, *Rh* Rhizome, *Ro* Root, *Se* Seeds, *Tu* Tuber, *WhPl* Whole plant, *E* Endemic, *IUCN* International Union for Conservation of Nature, *LC* Least Concern, *NT* Near threatened, *DD* Data Deficient, *HD* Habitats Directive Annexes

In terms of location, 122 (89.05%) medicinal species were only found to be growing in the wild. Eight (5.84%) species were only cultivated and 7 (5.11%) species were found both in the wild and cultivated (most often in gardens) (Table 4).

According to the ‘Regulations on the Proclamation and Protection of Protected Wild Plants, Animals, and Fungi’ that apply in Serbia, 55 of the total number of recorded plant species are protected, while 3 (*Agrimonia eupatoria* L.*, Lycopodium clavatum* L., and *Ruta graveolens* L.) are strictly protected [41]. The research showed that populations of *Gentiana lutea* L. were scarce due to excessive use of the rhizome, which was widely used by the local population to prepare a herb brandy known as ‘lincura’, a highly valued medicinal product.

### Preparation and use of medicinal plants

In addition to botanical information, Table 4 also contains information relating to the use of the plants in ethnomedicine: plant part/product, form of preparation, mode of application, diseases treated/number of respondents (ICPC-2), number of informants, and UV index.

**Fig. 4:**
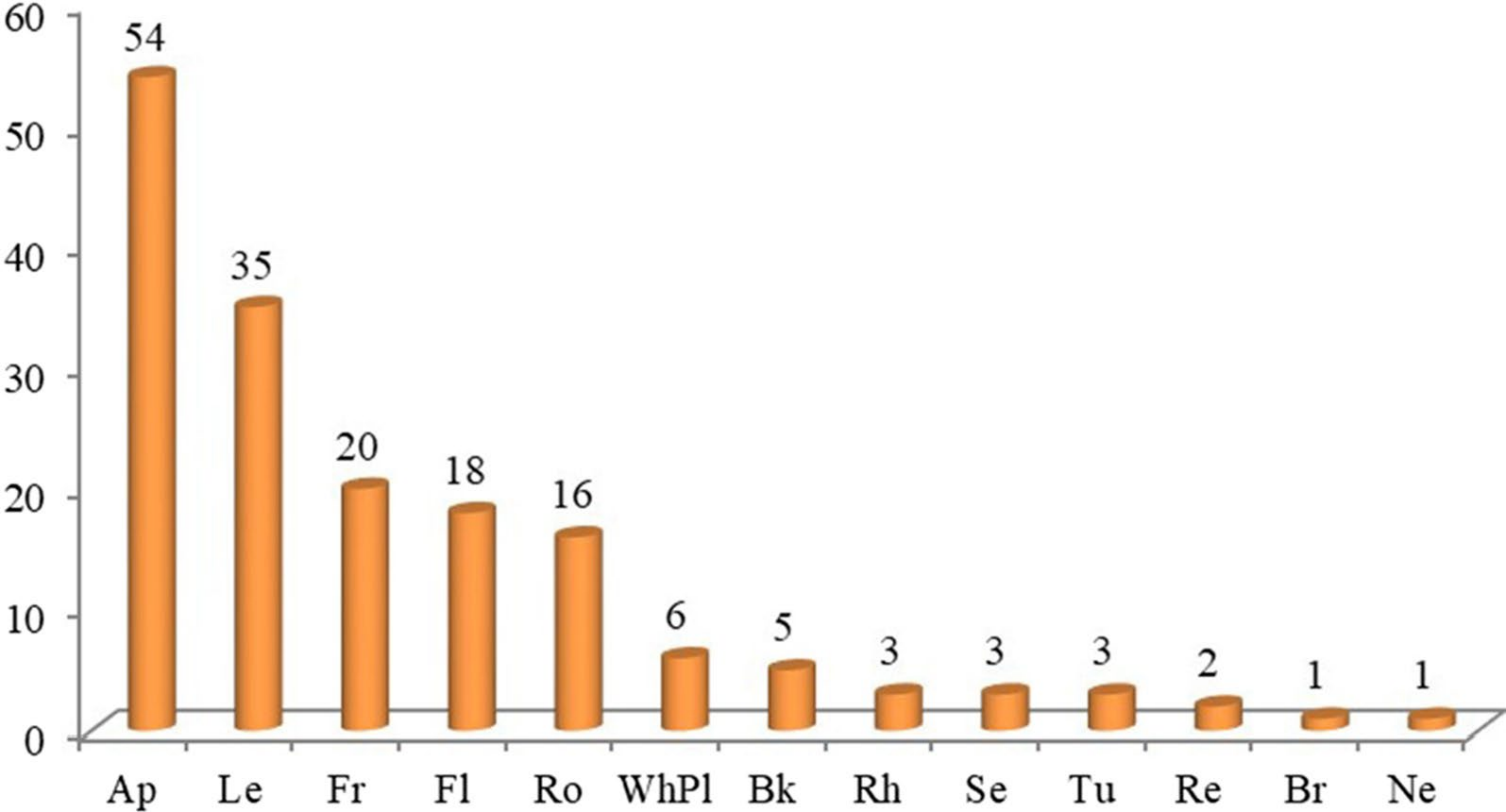
The most frequently used plant parts in the ethnomedicine of the study area

The plant parts most commonly used to make a variety of herbal preparations were the aerial parts (54 citations), leaves (35 citations), fruits (20 citations), flowers (18 citations), and roots (16 citations) (Fig. 4).

**Fig. 5:**
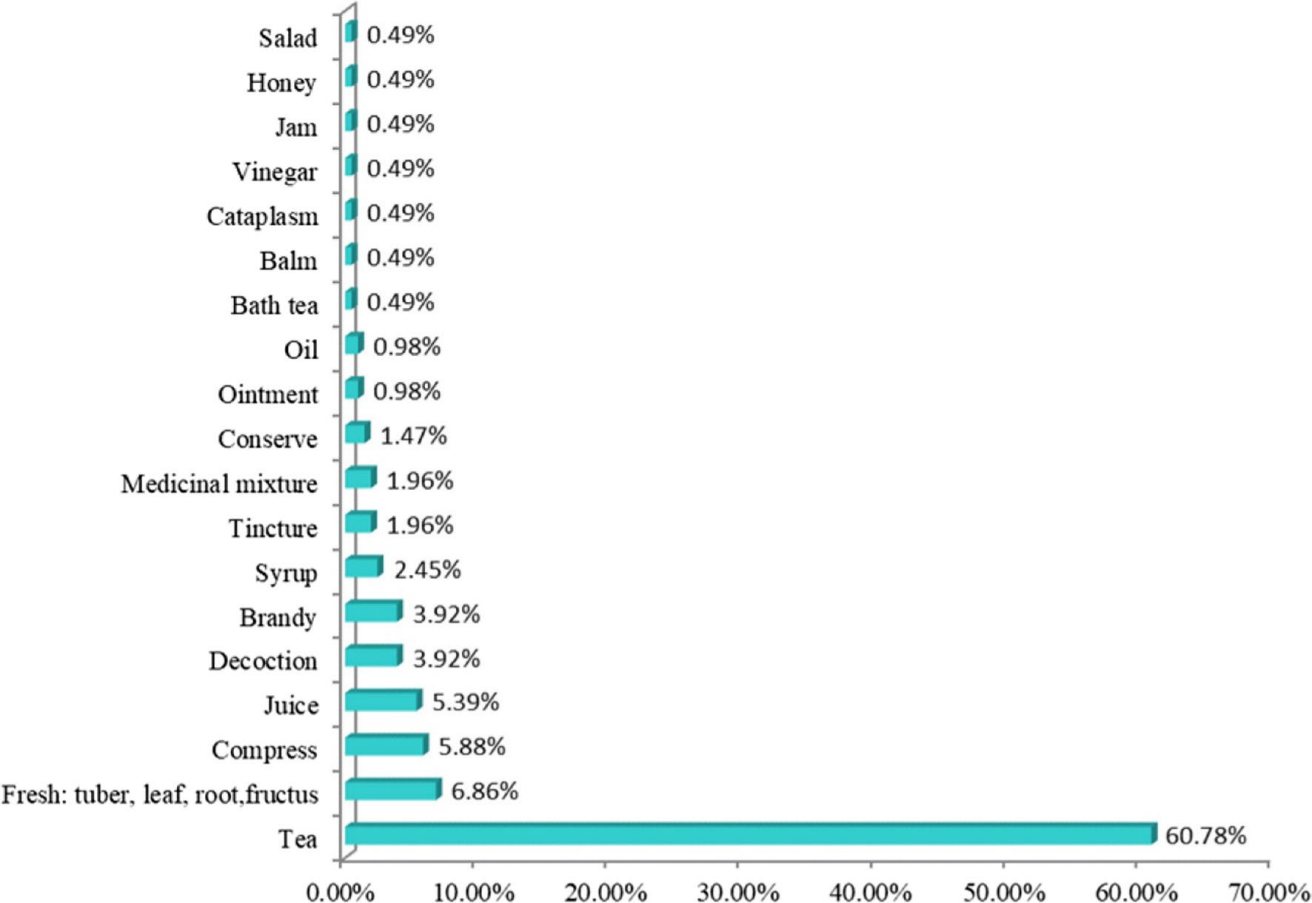
Form of preparation of medicinal plants

Of the recorded species, 102 were administered orally, 17 topically, and 18 both orally and topically. The most common preparation methods were teas (60.78%), consumption of fresh tubers, leaves, roots, and fructus (6.86%), compresses (5.88%), juices (5.39%), decoctions (3.92%), ‘travarica’ brandy (3.92%), and syrups (2.45%). The remaining preparation methods (tincture, ointment, oil, bath tea, balm, cataplasm, vinegar, jam, honey, and salad) were less frequently mentioned (Fig. 5). These findings are comparable to our previous work and the literature, where tea was also the most common method used to prepare herbal remedies in traditional medicine [2, 5, 6].

### Use value (UV)

**Fig. 6:**
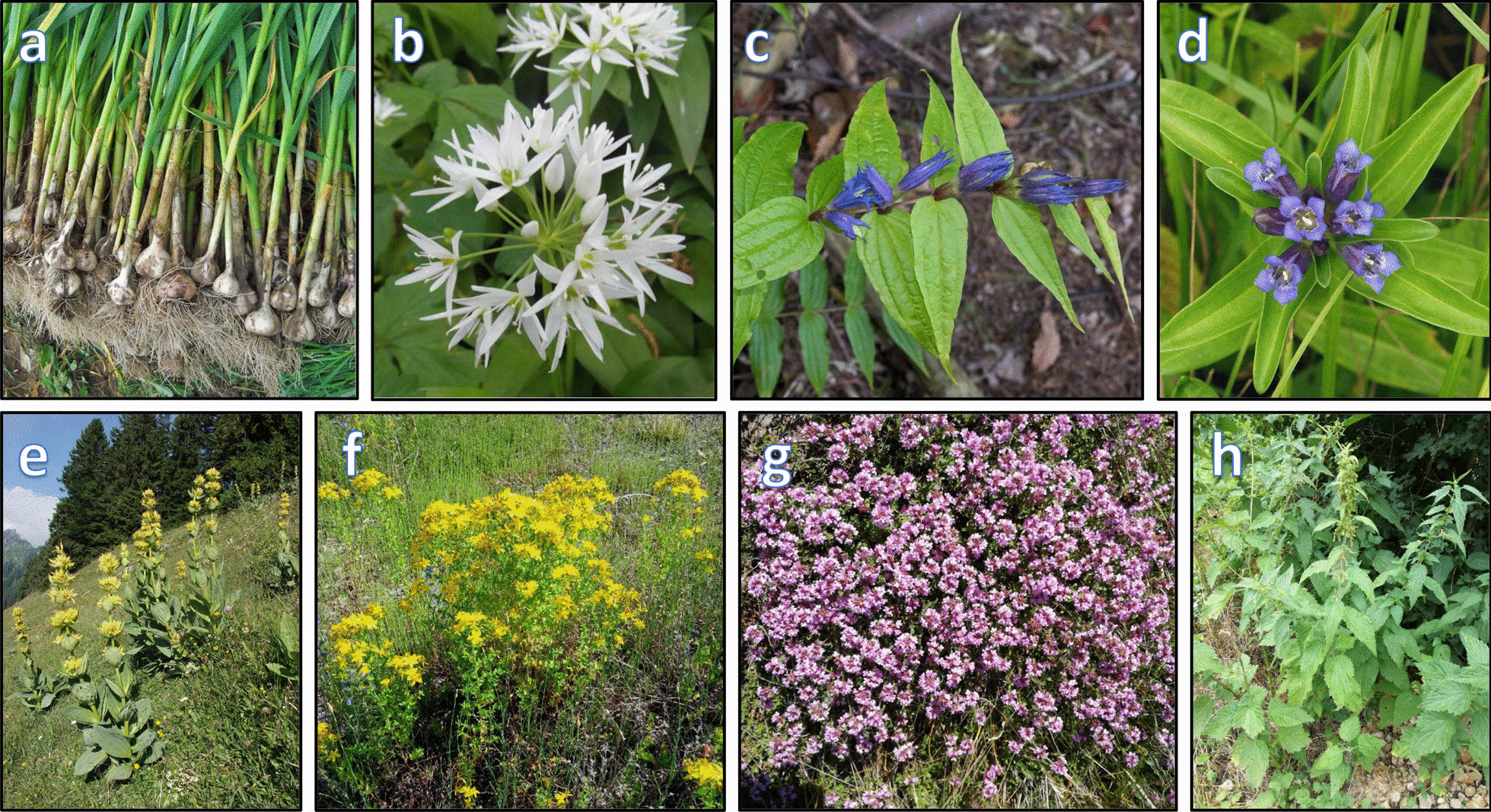
The plants with a maximum use value: **a*** Allium sativum,* **b*** Allium ursinum,* **c ***Gentiana asclepiadea,* **d** *Gentiana cruciata,* **e** *Gentiana lutea,* **f*** Hypericum perforatum,* **g** *Thymus serpyllum,* **h** *Urtica dioica* (All photographs by Jarić S.)

Use value is an essential tool for identifying highly valuable medicinal plants for potential further detailed pharmacological research. There is a strong correlation between use value and use reports for a certain plant. The high usage of reported medicinal plants implies a strong association and the reliance of local populations on the surrounding flora, particularly for the treatment of various diseases [42]. In this study, the use value ranged from 0.019 to 1 (Table 4). The highest use value (UV = 1) was reported for *Alium sativum* L., *Allium ursinum* L., *Gentiana asclepiadea* L., *Gentiana cruciata* L., *Gentiana lutea* L., *Hypericum perforatum* L., *Thymus serpyllum* L., and *Urtica dioica* L. (UV = 1) (Fig. 6; Table 4).

*Allium sativum* (garlic) and *A. ursinum* (wild garlic) were mainly used against high blood pressure and for health maintenance/preventive healthcare. Moreover, ethnobotanical research in other regions of Southeast Europe confirms the long-lasting, continuous tradition of using these plants in traditional medicine [2–6, 9, 11, 15, 43–45]. In addition, pharmacological research has shown that preparations made from *Allium ursinum* reduce hypertension and inhibit angiotensin-converting enzyme in vivo when tested on rats with spontaneous hypertension [46]. Similarly, Reuter established that the activity of *A. ursinum* as an angiotensin-converting-enzyme (ACE) inhibitor was considerably greater than that of *A. sativum* [47].

Species of the genus Gentiana (*G. asclepiadea, G. cruciata*, and *G. lutea*) were the most popular plant species among the population of the study area. The aerial parts of *G. cruciata* and *G. asclepiadea* were used in the preparation of ‘travarica’ herb brandy to treat stomach diseases and cancer, while the root of *G. lutea* was used to prepare ‘gentian brandy’ (lincura) to treat stomach problems and to improve digestion. Because of its healing properties and its wide variety of uses, the inhabitants of the Mt Stara Planina region considered *G. cruciata* to be a panacea (for nervousness, diabetes, killing parasites, liver disease, cholelithiasis, treating the prostate, etc.). The use of *G. asclepiadea* was similar: to treat malignancy, liver disease and jaundice, etc. An analysis of ethnobotanical literature showed that *G. cruciata, G. asclepiadea*, and *G. lutea* have similar uses in other mountainous regions in the Western Balkans [5, 8–10, 13, 14, 48, 49]. Phytochemical and biological research has shown that *G. lutea* contains physiologically important compounds that include iridoids, bitter constituents (gentiopicroside, amarogentine, swertiamarin, and sweroside), xanthones (gentisin, isogentisin), triterpenoid derivatives, and essential oils [50]. Secoiridoid glycosides that are most common in *G. lutea* contribute most to the anti-inflammatory, antitumour, hepatoprotective, wound healing, antifungal, antibacterial, and antioxidant activities of this species [51]. Also, xanthones and xanthone glycosides from *G. lutea* have been found to be potent inhibitors of monoamine oxidase in vitro [52]. In similar studies, it was found that methanolic extracts of the aerial part and root of *G. asclepiadea* exhibited hepatoprotective properties, while HPLC analysis revealed that the extracts were rich in gentiopicrin [53]. Experimental research also found that *G. cruciata* extracts significantly reduced DNA damage in liver cells caused by carbon tetrachloride. The antioxidant and antigenotoxic activities of this species confirm its therapeutic benefits and justify its use in traditional medicine [54]. Furthermore, phytochemical research into the dry extract of *G. cruciata* has shown the presence of tannins and polyphenols, which have antibacterial and antifungal properties. The native extract is highly active against *Staphylococcus aureus, Bacillus subtilis, Escherichia coli,* and *Candida albicans* [55].

In the study area, all the respondents mentioned the use of the species *Hypericum perforatum*, which they readily pick for their own needs but also to sell. The aerial part of this plant was used to prepare teas and oil, which were applied orally and topically. For the local population, its most important use was in the treatment of skin problems (burns, lacerations, skin injuries, etc.) and it is interesting to note that the medicinal properties of *H. perforatum* were known even in Hippocrates’ time, when it was farmed for its anti-inflammatory and wound healing properties [56]. Recent ethnobotanical studies conducted in other rural areas of the Balkan Peninsula have shown that *H. perforatum* is frequently used in ethnomedicine and is a well-known and favourite plant of the local population [2, 5, 6, 9, 13–15, 48, 57–59].

*Thymus serpyllum* was one of the ‘most significant’ medicinal plants, both ethnomedically and economically, for the current inhabitants of the Mt Stara Planina region. In the study area, the aerial part of this plant was mainly used for its antibacterial properties, to relieve cold symptoms, to treat respiratory diseases, influenza, and skin complaints, to regulate the menstrual cycle, to relieve heartburn, and as a sedative. In addition, numerous ethnobotanical studies have shown that this species has similar uses in the treatment of respiratory and gastrointestinal problems in other parts of the world, including the Western Balkans, where the study area lies [2, 5, 10, 13, 14, 48, 59–62].

In the area of 'Stara Planina' Nature Park, respondents mentioned the species *Urtica dioica* 103 times, most often to treat iron deficiency anaemia (40 times) and leukaemia (12 times) and for health maintenance/preventive healthcare (11 times). Similarly, this species is widely used in traditional medicine across the world and the health benefits of nettle are numerous: it improves circulation, heart health, and gastrointestinal health, it has antidiabetic properties, boosts the immune system, reduces inflammation, prevents kidney stones, aids in controlling blood sugar levels, helps detoxification, improves women's health, reduces the risk of prostate cancer, strengthens the bones, treats respiratory problems, and aids in pregnancy [63]. Phytochemical research has identified a wide range of biological properties that the leaves of this plant possess: antimicrobial, antiviral, antioxidant, anti-inflammatory, antiulcer, hypolipidemic, etc. [64]. This is a result of the presence of biologically active compounds: vitamins, amino acids, carotenes, fatty acids, terpenoids, fibres, and phenolic compounds [65].

The species *Hypericum perforatum* and *Urtica dioica* occupy an important place in Serbian traditional medicine, as well as in common beliefs. It is thus believed that on St. George's Day (6th May) you should take a bath in water which nettles have been soaked in. This will aid your general health. For St. John's Wort, also known in folk medicine as Virgin Mary grass, there is the belief in the south of Serbia that it got its spots after drops of water fell from the hands of the Virgin Mary onto its leaves [66].

A very popular species among the local population was *Sempervivum tectorum* L. (UV = 0.980), which was used to treat skin, ear, and cardiovascular problems. It was also cultivated as a decorative plant in yards and was often found on the roofs of houses because of the folk belief that it protects the house and household from lightning. Ethnobotanical studies conducted in the field of the distribution of *Sempervivum tectorum* show that this species is used for the same or similar health problems elsewhere [2, 5, 6, 8, 9, 58, 67–69].

Twenty-nine respondents (56.9%) from the study area mentioned the species *Hylotelephium spectabile* (Boreau) H. Ohba, which was grown in gardens, primarily for its medicinal properties (for the treatment of skin diseases), but also as an ornamental plant. By reviewing the ethnobotanical literature, we found that this species is mentioned in a small number of ethnobotanical studies [8, 13, 70–72], which is why it needs to be examined in more detail from a phytochemical and pharmacological point of view.

A special feature of this region was the use of the plant *Sambucus ebulus* L., which was cited eight times in the treatment of malignant diseases. In particular, respondents emphasised that one teaspoon of boiled jam should be consumed in the morning, before breakfast. This species is also used in other parts of the Western Balkans, but in the treatment of musculoskeletal disorders [6, 73].

## Informant consensus factor (ICF)

Analysis of ICF values showed that there was high homogeneity when it came to respondents' statements on the use of medicinal species within certain categories of diseases. The study included 2783 use reports for all taxa, referring to 138 diseases in 16 categories of health disorders (Table 5).

**Table 5:** Informant Consensus Factor values for ICPC-2 ailment categories

No	ICPC-2 categories	Ailments	Number of taxa (Nt)	Use reports (Nur)	ICF
1	S—Skin	S03 Warts (24); S10 Boil/carbuncle (26); S11 Skin infection post-traumatic (11); S13 Animal/human bite (4); S14 Burn/scald (62); S18 Laceration/cut (5); S19 Skin injury other (68); S20 Corn/callosity (1); S21 Skin texture symptom/complaint (10); S29 Skin symptom/complaint other (5); S76 Skin infection other (84); S77 Malignant neoplasm of skin (13); S79 Neoplasm skin benign/unspecified (1); S87 Dermatitis/atopic eczema (5); S96 Acne (2); S97 Chronic ulcer skin (63); S99 Skin disease, other (28);	22	412	0.95
2	B—Blood, Blood Forming Organs and Immune Mechanism	B02 Lymph gland(s) enlarged/painful (1); B73 Leukaemia (13); B80 Iron deficiency anaemia (94); B87 Splenomegaly (2); B99 Blood/lymph/spleen disease other (3);	7	113	0.95
3	L—Musculoskeletal	L20 Joint symptom/complaint NOS (11); L72 Fracture: radius/ulna (15); L73 Fracture: tibia/fibula (15); L74 Fracture: hand/foot bone (15); L75 Fracture: femur (15); L76 Fracture: other (15); L88 Rheumatoid/seropositive arthritis (11);	10	103	0.91
4	R—Respiratory	R01 Pain respiratory system (176); R05 Cough (129); R06 Nosebleeds/epistaxis (4); R07 Sneezing/nasal congestion (9); R25 Sputum/phlegm abnormal (3); R29 Respiratory symptom/complaint oth. (2); R72 Strep throat (9); R77 Laryngitis/tracheitis acute (4); R78 Acute bronchitis/bronchiolitis (49); R79 Chronic bronchitis (11); R80 Influenza (61); R95 Chronic obstructive pulmonary dis (5); R96 Asthma (28);	44	490	0.90
5	A—General and Unspecified	A01 Pain general/multiple sites (2); A03 Fever (8); A04 Weakness/tiredness general (10); A10 Bleeding/haemorrhage NOS (8); A11 Chest pain NOS (2); A70 Tuberculosis (6); A78 Infectious disease other/NOS (43); A79 Malignancy NOS (114); A80 Trauma/injury NOS (89); A92 Allergy/allergic reaction NOS (2); A98 Health maintenance/prevention (186);	51	470	0.89
6	D—Digestive	D01 Abdominal pain/cramps general (33); D02 Abdominal pain epigastric (5); D03 Heartburn (13); D07 Dyspepsia/indigestion (45); D08 Flatulence/gas/belching (11); D11 Diarrhoea (50); D12 Constipation (18); D13 Jaundice (30); D14 Haematemesis/vomiting blood (2); D16 Rectal bleeding (2); D18 Change faeces/bowel movements (5); D19 Teeth/gum symptom/complaint (1); D20 Mouth/tongue/lip symptom/complt. (4); D70 Gastrointestinal infection (17); D74 Malignant neoplasm stomach (4); D75 Malignant neoplasm colon/rectum (4); D77 Malig. neoplasm digest other/NOS (1); D85 Duodenal ulcer (7); D86 Peptic ulcer other (8); D87 Stomach function disorder (121); D94 Chronic enteritis/ulcerative colitis (15); D96 Worms/other parasites (5); D97 Liver disease NOS (34); D98 Cholecystitis/cholelithiasis (43);	58	478	0.88
7	K—Cardiovascular	K01 Heart pain (3); K07 Swollen ankles/oedema (1); K74 Ischaemic heart disease w. angina (1); K77 Heart failure (46); K79 Paroxysmal tachycardia (4); K80 Cardiac arrhythmia NOS (4); K85 Elevated blood pressure (76); K95 Varicose veins of leg (39); K96 Haemorrhoids (46);	29	220	0.87
8	H—Ear	H01 Ear pain/earache (8); H03 Tinnitus, ringing/buzzing ear (2); H70 Otitis externa (6); H71 Acute otitis media/myringitis (6);	4	22	0.86
9	T—Endocrine/Metabolic and Nutritional	T03 Loss of appetite (10); T08 Weight loss (1); T72 Benign neoplasm thyroid (1); T81 Goitre (2); T85 Hyperthyroidism/thyrotoxicosis (1); T86 Hypothyroidism/myxoedema (2); T90 Diabetes non-insulin dependent (72); T92 Gout (5); T93 Lipid disorder (65);	24	159	0.85
10	X—Female Genital	X01 Genital pain female (23); X02 Menstrual pain (5); X06 Menstruation excessive (4); X07 Menstruation irregular/frequent (22); X08 Intermenstrual bleeding (6); X11 Menopausal symptom/complaint (16); X29 Genital symptom/complt female oth. (40); X78 Fibromyoma uterus (7);	19	123	0.85
11	U—Urological	U01 Dysuria/painful urination (1); U02 Urinary frequency/urgency (5); U08 Urinary retention (33); U13 Bladder symptom/complaint other (5); U14 Kidney symptom/complaint (12); U70 Pyelonephritis/pyelitis (2); U71 Cystitis/urinary infection other (8); U72 Urethritis (8); U80 Injury urinary tract (4); U95 Urinary calculus (14);	33	92	0.65
12	W—Pregnancy, Childbearing, Family Planning	W15 Infertility/subfertility (7); W19 Breast/lactation symptom/complaint (2); W82 Abortion spontaneous (1); W83 Abortion induced (1); W91 Uncomplicate labour/delivery still (1);	5	12	0.64
13	P—Psychological	P01 Feeling anxious/nervous/tense (42); P06 Sleep disturbance (5); P12 Bedwetting/enuresis (1); P74 Anxiety disorder/anxiety state (5);	20	53	0.63
14	Y—Male Genital	Y06 Prostate symptom/complaint (7); Y08 Sexual function sympt./complt.(m) (1); Y10 Infertility/subfertility male (2); Y73 Prostatitis/seminal vesiculitis (4); Y85 Benign prostatic hypertrophy (6);	9	20	0.58
15	F—Eye	F70 Conjunctivitis infectious (1); F73 Eye infection/inflammation (2);	2	3	0.50
16	N- Neurological	N01 Headache (6); N19 Speech disorder (2); N28 Limited function/disability (n) (2); N88 Epilepsy (3);	10	13	0.25

The highest ICF value was recorded in the categories of skin diseases (S; ICF = 0.95) and Blood, Blood Forming Organs, and Immune Mechanism (B; ICF = 0.95). The homogeneity of statements was also high in the following categories of diseases: Musculoskeletal (L; ICF = 0.91), Respiratory (R; ICF = 0.90), General and Unspecified (A; ICF = 0.89), Digestive (D; ICF = 0.88), Cardiovascular (K; ICF = 0.87), Ear (H; ICF = 0.86), Endocrine/Metabolic and Nutritional (T; ICF = 0.85), and Female Genital (X; ICF = 0.85) (Table 5). The high ICF values found in most of the categories of medicinal uses can be explained by the fact that there was high homogeneity of consensus among informants on the therapeutic uses of a set of species and on their efficacy [74]. The lowest ICF value was in the neurological category (N; 0.25).

In the skin category, respondents mentioned 18 different types of skin diseases and problems. These were treated using 22 plant species, which were cited a total of 412 times. They were most commonly used in the treatment of skin infections (S76—84 times), skin injuries (S19—68 times), and chronic skin ulcers (S97—63 times). They were applied externally and the respondents most often mentioned *Hypericum perforatum* (79 times), *Plantago lanceolata* L., and *Plantago major* L. (72 times each).

In the Blood, Blood Forming Organs, and Immune Mechanism category, respondents listed five different types of diseases. These were treated using seven plant species, which were cited a total of 113 times and were most often mentioned in the treatment of iron deficiency anaemia (B80—94 times) (Table 2). In this category of health problems, the most commonly mentioned plant species were *Urtica dioica* (52 times)*, Cornus mas* L. (41 times), and *Rubus fruticosus* L. (13 times), which were also an integral part of the diet of the inhabitants in the study area. The fruits (*Conus mas* and *Rubus fruticosus*) and aerial parts (*Urtica dioica*) were used and taken orally.

The second highest ICF was found in the musculoskeletal category. Ten types of plants were used in the treatment of bone fractures, joint pain, and rheumatic pain and respondents cited them 103 times. The most important plant for the treatment of musculoskeletal problems was *Symphytum officinale* L. (cited 84 times). Its root was used to make ointment (the ground root is cooked in milk and left to stand overnight; the dressing is changed every second day over a period of two months) and applied externally in the form of a compress. This plant is highly valued in Serbian ethnomedicine [5, 73, 75, 76]. In their phytochemical and pharmacological research, Sowa et al. determined that *Symphytum officinale* root extract contains allantoin and phenolic acids (rosmarinic, p-hydroxybenzoic, caffeic, chlorogenic, and p-coumaric acids), which indicates its strong antioxidant potential and a beneficial effect on human skin fibroblasts [77]. Also, allantoin is considered to play an important role in the healing of wounds and bones in the case of fractures. It has been claimed that allantoin is the active ingredient in *Symphytum officinale* and is responsible for triggering cell division and wound healing, as well as promoting conjunctive tissue, bone, and cartilage growth [78]. The results of this and similar studies justify the use of *S. officinale* in ethnomedicine.

The third highest ICF was found for Respiratory problems, where respondents cited the use of 44 plant species mentioned a total of 490 times for the treatment of 13 different types of respiratory complaint. Plants were mostly used in the treatment of respiratory system pain (R01—176 times) and coughs (R05—129 times), while *Thymus serpyllum* was the most commonly mentioned plant (63 times). Also important for the treatment of respiratory complaints in this region were *Tilia cordata* Mill. (mentioned 39 times), *Mentha x piperita* L. (mentioned 39 times), and *Satureja montana* L. (37 times), which, like *Thymus serpyllum*, were taken orally, in the form of tea.

### Novel ethnobotanical reports

Comparative analysis of the obtained results with the results of other ethnobotanical studies conducted in Serbia and the Balkans found that the species *Agrimonia eupatoria, Gentiana asclepiadea, Geranium robertianum, Origanum vulgare, Oxalis acetosella, Polygonum aviculare,* and *Stachys officinalis* are mentioned for the first time as being used in the treatment of jaundice. The aerial parts of *Urtica dioica* and the leaves of *Scrophularia nodosa* are also mentioned for the first time in the treatment of goitre. Other recent findings have shown that *S. nodosa* is used in traditional medicine in the Deliblato Sands region (Serbia), but it is the roots and shoots that are used to treat cancer, goitre, rabies, skin ulcers, haemorrhoids, rashes, and eczema, while the seeds are used as an anthelmintic [79]. In the south-western part of the Sharr Mountains (North Macedonia), the aerial parts of *S. nodosa* are topically applied to treat tuberculosis [58], while in the Prokletije region it is used in the treatment of breast cancer [13]. The biological screening of *S. nodosa* extract and its fractions has revealed that this species may be a good source of antimicrobial, antioxidant, and analgesic compounds [80].

*Achilea clypeolata* is a Balkan endemic species and is highly valued among the population of the study area. It is used in the ethnomedicine of the region to treat respiratory system pain, bleeding/haemorrhages, and symptoms of female genital diseases; this is the first report on this species being used to treat these health issues in the Balkans and Serbia. However, data does already exist on the use of this species in Serbia (Suva planina and Rtanj), but to treat kidney problems, improve appetite, soothe coughs [5], and treat diabetes [15].

*Carlina acaulis* is used in the Stara Planina region to treat diabetes, gout, and urinary calculus. The ethnomedicinal properties of this species are familiar to the peoples of the Balkans, but in different regions it has different uses. In the region of central Serbia (Kopaonik), it is used to treat skin complaints, ulcers, acne, eczema, and wounds [2], while in Bosnia it is used to treat skin injuries [81], influenza, high temperatures, headaches, urinary tract infections, worm infections, increased diuresis, renal ailments, and inflammation of the throat [57]. In Montenegro (Prokletije), *C. acaulis* roots are used to treat gastritis, dyspepsia, and bile duct disorders, and when applied externally, to treat dermatoses, wounds, and ulcerations [13]. In the region of North Macedonia (Sharr Mountains), the flower, root, and seed are used in the treatment of acne and eczema [82]. In Bulgaria, *Carlina acanthifolia* root is used as a diuretic and urogenital anti-inflammatory [83].

*Carlina vulgaris* is used in the ethnomedicine of Mt Stara Planina to treat haemorrhoids, which is the first such report for this species. Phytochemical research has shown that *C. vulgaris*, one of the least studied species of the genus, is rich in polyphenols and minerals, especially populations that grow in uncontaminated areas, which contain more chlorogenic acid and exhibit greater antioxidant activity [84]. It has also been found to have a protective effect against H2O2-induced oxidative stress in human skin fibroblasts [85] and the antioxidant and cytotoxic potential of *C. vulgaris* against human colorectal adenocarcinoma has been determined [86].

*Nasturtium officinale* is mentioned in the treatment of acne, which is also the first such report for the Balkans, and therefore for Serbia, too. However, this species was mentioned as far back as the eleventh century, in 'The Trotula', the first textbook on aesthetic medicine, written by Trota of Salerno, where it was noted for its anti-ageing properties [87]. Chemical analyses of *N. officinale* have revealed the presence of alkaloids, flavonoids, saponins, terpenoids/steroids, protein, essential and volatile oils, glycosides, tannins, folic acid, vitamins, and elements [88]. Also, pharmacological research has determined the hypolipidemic, anti-inflammatory, hepato-renal protective, antidiabetic, antioxidant, anticancer, antimicrobial, dermatological, antigenotoxic, anti-urolithiatic, and antigenotoxic properties of this species [88].

*Heracleum sphondilium* is mentioned for the treatment of epilepsy, which is a new finding for Serbia and the Balkans. Previous ethnobotanical research conducted in the Suva planina area (Serbia) revealed the use of this species to treat rheumatoid arthritis [5]. Data exists that shows the stem and seeds of *Heracleum persicum* Desf. ex Fischer are used for the treatment of epilepsy in Iranian ethnomedicine. Biological research has shown that *H. sphondylium* exhibits antimicrobial, cytotoxic, and vasorelaxative activity [89].

In the ethnomedicine of the study area, *Filipendula ulmaria* (L.) is used in the treatment of uterine fibroids, heartburn, and peptic ulcers, which is also new data for the use of this species. In the region of Timok and the Svrljig Mountains, this plant is used to treat musculoskeletal (arthritis), digestive (liver disease), urological (urination), endocrinal, metabolic, and nutritional (anti-obesity) issues [73], while in the Kopaonik area it is used in the treatment of rheumatism [2]. In Bosnia, it is drunk in the form of a tea to treat the common cold [81], while in Montenegro (Prokletije) it is used in the treatment of coughs, bronchitis, fevers, colds, and rheumatism of the joints and muscles [13]. Phytochemical research has revealed the presence of several active compounds, mainly phenolic acids, flavonoids, tannins, and terpenoids. Salicylic acid and its derivatives are the most important compounds found in the essential oil and extracts from different parts of the plant. Pharmacological analyses have determined the analgesic, anti-arthritic, antimicrobial, anti-inflammatory, anticancer, antioxidant, anticoagulant, immunomodulatory, gastro-protective, and hepatoprotective activity of *F. ulmaria* [90, 91].

*Sambucus ebulus* being used to treat malignancy is a new finding for Serbia and the Balkans. Generally speaking, this plant is very well known to the peoples of the Balkans and, according to ethnobotanical data, is widely used. In other parts of Serbia, this species is used to treat arthritis, gout [73], and gallstones [76], as an anti-rheumatic and diuretic, to cleanse and induce perspiration, for the removal of warts [79], and as an antidote to snake bites (juice from the ground leaf is applied to the bite) [2, 10]. In the Kosovo region, *S. ebulus* is used as an anti-rheumatic, for menstrual pains and regulation of the menstrual cycle, urinary inflammations [9], constipation, and respiratory disorders [92]. Menković et al. [13] described the use of this species in the Prokletije region (Montenegro) to treat gout, rheumatic complaints, and oedema, while Pieroni et al. [59] note that it is topically applied to treat snake bites in Western Macedonia (Upper Reka Valley, Mount Korab). In Bosnia and Herzegovina, *S. ebulus* has traditionally been used against pulmonary ailments, coughs, and hoarseness, to treat increased diuresis and kidney and bladder ailments, for restlessness and blood purification, to reduce blood sugar levels, and against rheumatism and fluid retention [6, 57, 93]. Also, ethnobotanical research has shown that in Eastern Albania *S. ebulus* is applied externally to treat bruises, wounds, and cold sores [94, 95].

Phytochemical and pharmacological research has shown that crude methanol extract of *S. ebulus* leaves has remarkable wound healing properties. This may be the result of the synergistic effect of the constituents present in the methanolic extract. Namely, *S. ebulus* methanol extract at one per cent concentration has been found to exhibit considerable wound healing activity in both linear and circular excision animal models. This property, which is linked to 'quercetin 3-O-glucoside' as a flavonoid derivative, is supported by a histopathological examination of wound models [96]. Experimental research has shown that the extracts of the flowers and leaves of *S. ebulus* possess anti-inflammatory potential by effectively suppressing the biosynthesis of tumour necrosis factor (TNF-α), interleukin 1-α (IL1-α), and interleukin 1-β (IL1-β) [97] *S. ebulus* fruit extract also exhibits anti-inflammatory properties through significant nitric oxide removal activity (inflammation indicators) [98]. Saravi et al. [99] investigated the antitumour properties of *S. ebulus* and found that ethyl acetate extract of this species exhibits high cytotoxicity against human hepatocarcinoma and human colon carcinoma cell lines. In addition, experimental research has shown that *S. ebulus* also possesses antioxidant, antimicrobial, wound healing, antigiardial, scolicidal, analgesic, antidepressant, and neuroprotective properties [100]. It is clear that *S. ebulus* has a wide range of therapeutic effects, which is why there is a need for further research to assess its effectiveness and how safe it is. In general, the raw fruits of this plant are considered poisonous, but other parts of the plant can also be toxic if used in excess: they can induce vomiting, especially in children [100]. In this regard, respondents who stated that they used this species emphasised the need to limit consumption (it must be measured out, 1 teaspoon in the morning).

Data on the use of *Sanicula europaea* for health maintenance/preventive health care and treatment of strep throat and gout in the study area is new for the Balkan region. In general, there is not much data on the use of this species in ethnobotanical literature. In the Balkans, in addition to the study area (Mt Stara Planina), it has also been recorded as a ritual plant and a plant that solves 'love disorders' in eastern Serbia (Timok and Svrljig Mountains) and as a plant used to heal wounds and to treat skin problems and bleeding in Montenegro [13] and Bosnia and Herzegovina [62]. Pharmacological research has established the in vitro anti-inflammatory activity of *S. europaea* roots due to the reduced production of IL-8 and E-selectin after stimulation with TNF-α and LPS [101].

Our research also revealed interesting new findings for Serbia and the Balkans for the following species: *Tanacetum vulgare* (induced abortion), *Dioscorea communis* (gout), and *Consolida regalis* (prostatitis). The importance of these new reports on the specific uses of certain plants in Serbia and the Balkans as a whole lies in the chance to find new uses for medicinal plants and to discover new herbal remedies.

### Conservation status of medicinal plants

Plants are a very important part of any ecosystem because they form its physical structure and are essential for the functioning of the planet's atmosphere. However, many plant species, especially those intended for a particular purpose, such as being used for medicinal purposes, are directly threatened by over-exploitation and the impact of invasive species, as well as climate change posing an increasing threat [102]. For these reasons, plants in the priority conservation class in Serbia are protected by national legislation and some international regulations such as The Convention on Biological Diversity, the Bern Convention (The Convention on the Conservation of European Wildlife and Natural Habitats), CITES (The Convention on International Trade in Endangered Species of Wild Fauna and Flora), and the Habitat Directive (Council Directive 92/43/EEC on the Conservation of Natural Habitats and of Wild Fauna and Flora). This study identified the endemic species *Achillea clypeolata* Sm. (Balkan endemic), used for medicinal purposes. According to the IUCN list (https://www.iucnredlist.org/), 15 plant species are in the category of Least Concern (LC). However, the species *Anacamptis morio* (L.) R.M. Bateman, Pridgeon & M.W.Chase is in the category of Near Threatened (NT) and also on the CITES list (Annex II), while the Data Deficient (DD) category includes the species *Malus sylvestris* Mill. The list of the Habitat Directive (HD; Annex V) includes the species *Gentiana lutea*. The identified medicinal species with their related degree of protection are shown in Table 4.

The biggest threat to protected species in the study area is the impact of the anthropogenic factor. Namely, people picking certain plants in excess, e.g. *Gentiana lutea*, has contributed to a significant reduction in populations of these species. A key measure that should be regularly undertaken in the study area would be to educate the local population on the importance of medicinal plants.

### Medicinal plants as foodstuffs and a valuable resource for the herbal market and ecotourism

The Mt Stara Planina region is a rural one with preserved traditional village-type settlements, human activities, and local agricultural products, which are a recognisable brand and identity of this part of south-eastern Serbia. The region's inhabitants are engaged in livestock farming and traditional agriculture, with their primary task being to provide sufficient food [29]. In order to improve both their standard of living and to foster the economic development of this rural area, the local population is also involved in picking forest fruits, mushrooms, and wild medicinal plants, which are then purchased by local traders.

**Fig. 7:**
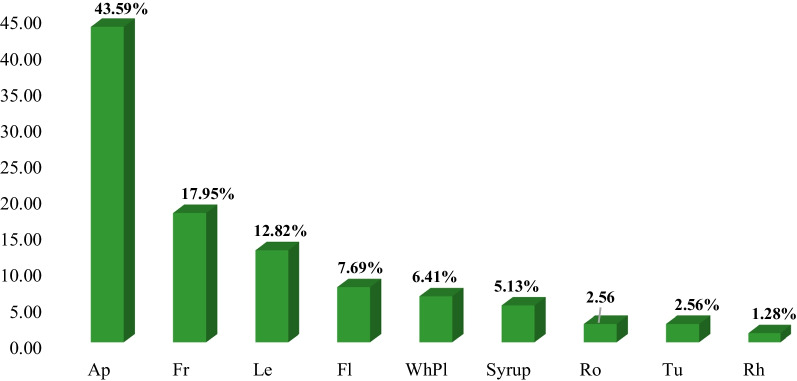
Percentage use of plant parts in the diet of the local population

**Fig. 8:**
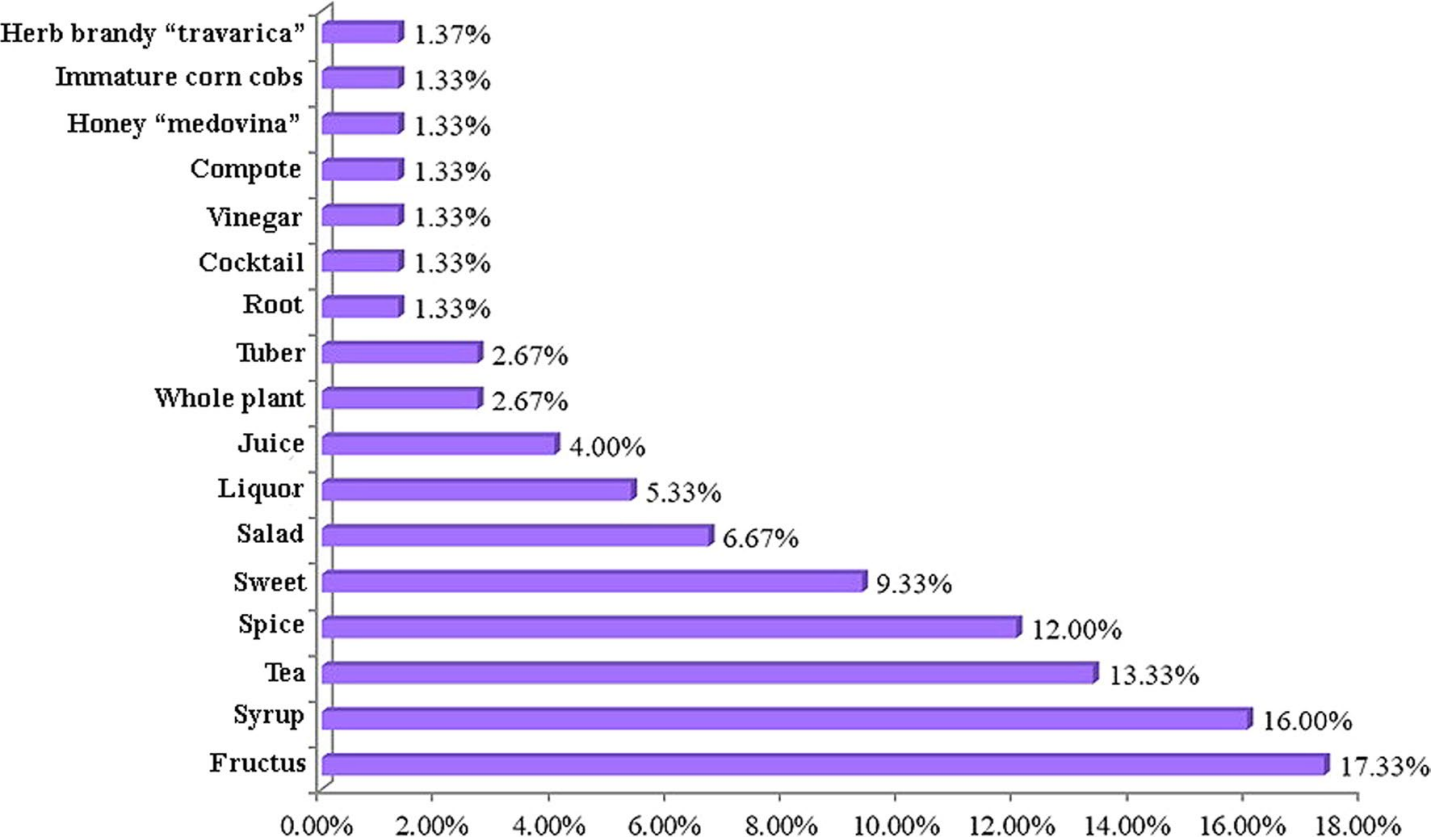
Frequency of medicinal plant preparation forms

In regard to the use of medicinal plants as foodstuffs, the obtained results revealed that the local population uses 36 species of plants for food. These are prepared in 18 ways, with the aerial parts (43.59%), fruits (17.95%), and leaves (12.82%) used most commonly (Fig. 7). Medicinal plants are predominantly consumed in the form of fresh fruits (17.33%), syrups (16.00%), teas (13.33%), and spices (12.00%), but also in the form of sweets (9.33%) and salads (6.67) and as liquors (5.33%) and juices (4.00%) (Table 6; Fig. 8).

**Table 6:** Economically important medicinal plants and medicinal plants as foodstuffs

Plant species	Economically important plants/for sale	Used as a foodstuff
*Achillea clypeolata* Sm	Ap (dried)	
*Achillea millefolium* L	Ap (dried)	
*Agrimonia eupatoria* L	Ap (dried)	
*Alchemilla vulgaris* L	Ap (dried)	
*Allium cepa* L	WhPl (young plants),Tuber	WhPl, Tu: Salad, spice
*Allium sativum* L	WhPl (young plants), Tuber	WhPl, Tu: Salad, spice
*Allium ursinum* L	Le (young; fresh or dried)	Le: salad, spice
*Artemisia absinthium* L	Ap (in flower)	Ap: liquor ‘travarica’
*Calendula officinalis* L	Ap, Fl	
*Capsella bursa-pastoris* Medik	Ap (dried)	
*Carlina acaulis* L		Ro (young plants)
*Centaurium erythraea* Rafn	Ap (dried)	
*Chelidonium majus* L	WhPl, Ap (dried)	
*Cichorium intybus* L	Ro, WhPl	
*Cornus mas* L	Fr, Syrup	Syrup, liquor
*Corylus avellana* L	Fr	Fr (cakes and cookies)
*Cydonia oblonga* Mill	Le (dried), Fr (fresh)	Fr (fresh, baked), sweet, juice, syrup
*Epilobium parviflorum* (Schreb.) Schreb	Ap (dried)	
*Equisetum* sp.	Ap (dried)	
*Fragaria vesca* L	Fr (fresh), Le (dried)	Fr (fresh), sweet, syrup, cocktail
*Fumaria officinalis* L	Ap (dried)	
*Galium verum* L	Ap (dried)	
*Gentiana asclepiadea* L	Ap (dried)	
*Gentiana cruciata* L	Ap (dried)	
*Gentiana lutea* L	Ro (dried)	
*Geranium robertianum* L	Ap (dried)	
*Hypericum perforatum* L	Ap (dried)	
*Hyssopus officinalis* L	Ap	
*Juglans regia* L	Fr	Fr, liquor ‘orahovača’’
*Juniperus communis* L	Fr (fresh)	
*Lythrum salicaria* L	Ap	
*Malus sylvestris* (L.) Mill	Fr	Vinegar
*Malva sylvestris* L	Fl, Le	
*Marrubium vulgare* L	Ap	
*Matricaria chamomilla* L		Tea
*Melilotus officinalis* (L.) Pall	Ap	
*Melissa officinalis* L	Ap	Syrup
*Mentha* × *piperita* L	Ap	Spice, tea
*Mentha pulegium* L	Ap	Spice, tea
*Mentha spicata* L	Ap	Spice, tea
*Morus nigra* L		Fr (fresh)
*Nasturtium officinale* R.Br		Ap—salad
*Origanum vulgare* L	Ap (dried)	
*Persicaria bistorta* Samp	Rh (dried)	
*Phaseolus vulgaris* L		Fr
*Plantago lanceolata* L	Le (dried)	
*Plantago major* L	Le (dried)	
*Polygonum aviculare* L	Ap (dried)	
*Primula veris* L	Ap	
*Prunus avium* (L.) L	Fr	Fr (fresh), compote, liquor, sweet
*Pulmonaria officinalis* L	Ap	
*Pyrus pyraster* (L.) Burgsd	Fr	Herb brandy ‘travarica’
*Rosa canina* L	Fr (fresh)	Jam, tea
*Rubus plicatus* Weihe & Nees	Fr (fresh)	Fr (fresh), sweet, juice, syrup
*Rubus idaeus* L	Le (dried), Fr (fresh)	Fr (fresh), juice, syrup
*Rumex patientia* L		In cooking: fresh leaves for soup or ‘sarmice’
*Salvia officinalis* L	Ap (dried)	
*Sambucus nigra* L	Fl (dried), Syrup	Fl: syrup, Fr: sweet
*Satureja montana* L	Ap (dri**e**d)	Tea, spice
*Sorbus domestica* L		Fr when the fruits have rotted
*Stachys officinalis* (L.) Trevis	Le (dried)	
*Taraxacum officinale* F.H.Wigg	WhPl	Fl: Sweet, honey ‘medovina’, Syrup, Le: salad
*Teucrium chamaedrys* L	Ap	
*Teucrium montanum* L	Ap	
*Thymus serpyllum* L	Ap	Syrup, tea, spice
*Tilia cordata* Mill	Fl	Tea
*Tilia platyphyllos* Scop	Fl	Tea
*Tilia tomentosa* Moench	Fl	Tea
*Tussilago farfara* L	Le (dried)	
*Urtica dioica* L	Le (dried), Se	Syrup, spice for dishes, for soup
*Vaccinium myrtillus* L	Fr (fresh), Syrup	Fr (fresh), syrup, sweet
*Vaccinium vitis-idaea* L	Fr (fresh), Syrup	Fr (fresh), syrup
*Zea mays* L		Immature corn cobs: roasted or boiled

*Ap* Aerial part, *Fr* Fruit, *Fl* Flower, *Le* Leaf, *Rh* Rhizome, *Tu* Tuber, *WhPl* Whole plant, *Ro* Root

In terms of how plant species are used as food, there are great similarities with neighbouring regions. For instance, the local population in Bulgaria gathers the aboveground parts of plants, e.g. *Urtica dioica, Rumex patientia,* and *Taraxacum officinale*, mainly during the spring and uses them as vegetables, while fruits are mostly gathered from woody and shrubby plants from the families *Rosaceae, Viburnaceae, Ericaceae*, and *Vitaceae* [103]. Nedelcheva et al. [103] highlight that the jams and jellies prepared from cornel (*Cornus mas*) have an astringent effect, while soup (or other meals) made from *U. dioica* (nettle) is generally used to strengthen the constitution especially after a long illness, to treat anaemia, and as a natural blood purifier, similar to in the Mt Stara Planina region. Ethnobotanical research conducted in eastern and south-eastern Albania, as well as in nearby villages located in North Macedonia, showed that the most commonly used wild plants included *Cornus mas* fruits, *Urtica dioica* and *Rumex patientia* leaves, *Origanum sp.* and *Sideritis sp.* aerial parts, and *Prunus cerasifera* fruits [104]. In south-eastern Romania, several taxa are used as foodstuffs, e.g. *Rumex, Malva sylvestris, Sambucus nigra* and *Urtica dioica*, with *U. dioica* clearly identified as a distinctive feature in the diet of Romanians [105].

Data relating to the use of plants to make alcoholic beverages is interesting: 'travarica' herb brandy (*Pyrus piraster*), liquors (*Artemisia absinthium, Cornus mas, Juglans regia, Prunus avium*), and cocktails (*Fragaria vesca*). Flavoured alcoholic drinks specific to certain regions are deeply rooted in the tradition of herbalism and are an important part of local culinary identity in Eastern Europe including the Balkan countries [106, 107].

**Fig. 9:**
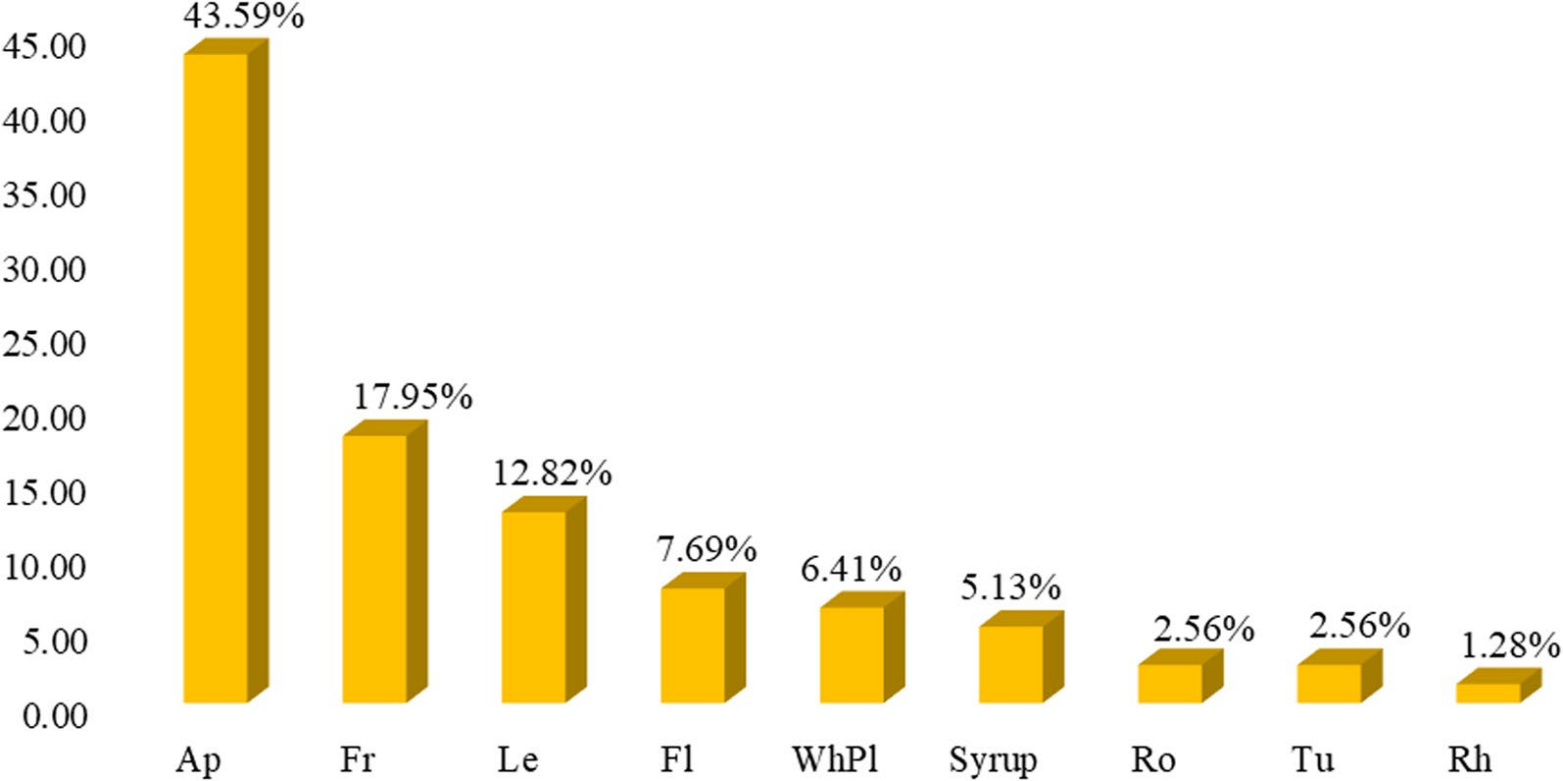
Medicinal plants, plant parts, and herbal products of commercial importance

Our research also found that 65 plant species are of economic significance for the local population in that they are traded (Table 6). This is particularly important given the current and potential contribution of these species to the local economies and their increased value to those harvesting them in the long term. Most often it is the aerial parts (43.59%), fruits (17.95%), and leaves (12.82%) that are harvested. They are bought and sold both fresh and dried (Fig. 9).

Based on the existing renewable resources of Mt Stara Planina, there is great potential to develop an area of significant economic activity, including the gathering and processing of forest berries and fruits, and the harvesting, processing, and final production of medicinal and aromatic plants. Traditionally, on St. John's Day (Ivanjdan, 24th June), there is an organised harvest of medicinal and aromatic plants on Mt Stara Planina.

In Serbia, the harvesting and trade of medicinal plants dates back to 1930, when 1,810 tons of medicinal plants were exported [108]. Recent data shows that in the period 2004–2016, 17,807,282.86 kg were exported and a profit of EUR 54,461,300.36 was made [109]. There are 110 companies that deal in the purchase and sale of medicinal plants in Serbia [110]. There is no precise data for the Mt Stara Planina region, but it is known that there are a number of family-run and local companies that are engaged in harvesting and buying up medicinal plants and thus have a positive impact on the development of the local community (e.g. **'**Stara Planina' Association for Medicinal Plants, 'Babin Nos' Association, Stanko Madić—Teas from Stara Planina, The Agricultural Estate of Dragiša Dimitrova from Temska—Medicinal Plants, Mushrooms, and Teas, etc.). The only company authorised by the 'Srbijašume' Public Enterprise to purchase forest fruits harvested from the Mt Stara Planina region is the 'Temac' agricultural cooperative owned by the Pavlović family from Temska near Pirot, which deals in the purchase and processing of products which are then sold to the German and Slovenian markets. To date, the local population that harvests the herbs which are then bought up usually receives inadequate financial compensation for the raw plant materials. It is clear that medicinal plants as a renewable natural resource, their processing, and final production could enable the creation of more employment opportunities for a large number of workers involved in different occupations. In addition, it could facilitate the relocation of manufacturing plants from industrial centres with a tendency to establish smaller plants in rural areas [111] such as the Mt Stara Planina region.

Ecotourism based on medicinal plant resources is becoming increasingly popular, reflecting the growing demand for medicinal plants as a field of alternative medicine. 'Stara Planina’ Nature Park, declared as such in 1997, represents the main tourism potential of the Pirot municipality. However, ecotourism in this region is still at an early stage, with rural tourism poorly organised and insufficiently linked to other forms of tourism (e.g. hiking, skiing, hunting and fishing tourism, etc.) [111].

## Conclusion

The ethnobotanical research conducted in the Mt Stara Planina region is an attempt to update our knowledge on the diverse therapeutic applications of different medicinal plants, which are clearly of great importance in the lives of the local population. The knowledge of the medicinal properties of the plants and how they are used by the local population is a result of the specific geographical location of the study area, high biodiversity, ethnic and cultural differences, and folk traditions that have evolved over the centuries. Traditionally, plants have been used to treat a wide range of health problems. This study has presented data relating to the traditional use of medicinal plants in human medicine (136 plant species and 1 lichen species). Eight plant species had a maximum use value (UV = 1). The highest ICF value was observed in the categories of Skin and Blood, Blood Forming Organs, and Immune Mechanism. Our research, focussing on Serbia and the Balkans, revealed new ways of using certain medicinal plants from the Mt Stara Planina region—*Agrimonia eupatoria, Gentiana asclepiadea, Geranium robertianum, Origanum vulgare, Oxalis acetosella, Polygonum aviculare* and *Stachys officinalis* to treat jaundice, *Urtica dioica* and *Scrophularia nodosa* to treat goitre, the endemic *Achilea clypeolata* to treat respiratory system pain, bleeding/haemorrhages, and symptoms of female genital diseases, *Carlina acaulis* to treat diabetes, gout, and urinary calculus, *Carlina vulgaris* to treat haemorrhoids, *Nasturtium officinale* for acne, *Heracleum sphondilium* for epilepsy, *Filipendula ulmaria* to treat uterine fibroids, heartburn, and peptic ulcers, *Sambucus ebulus* to treat malignancy, and *Sanicula europaea* for health maintenance/preventive health care and the prevention of strep throat and gout. New findings for the following species are also significant: *Tanacetum vulgare* (inducing abortions), *Dioscorea communis* (gout), and *Consolida regalis* (prostatitis). Therefore, the richness of the biodiversity and the unique biocultural heritage of the local people from the Mt Stara Planina region are highly valued. This is reflected by measures that have been undertaken with the formation of the protected ‘Stara Planina’ Nature Park for biodiversity conservation. However, further efforts in terms of the conservation of human Traditional Ecological Knowledge diversity and cultural heritage are also necessary. This study has documented traditional knowledge on the use of medicinal plants in ethnomedicine, which should be preserved to prevent it from being lost and forgotten. The importance of medicinal plants used as foodstuffs and their significance for the local market and exports as well as ecotourism should not be ignored either. Furthermore, this research proves that the Western Balkans is a unique area, suitable for further, detailed studies on traditional medical ethnobotany and phytotherapy, which is in line with its biological, ethnic, and cultural diversity.

## Data Availability

All the necessary data collected for this study was analysed and included in this manuscript.

## Abbreviations

A: General and unspecified
ACE: Angiotensin-converting enzyme
Ap: Aerial part
B: Blood, Blood Forming Organs and Immune Mechanism
Bk: Bark
Br: Branches
C: Cultivated species
CITES: The Convention on International Trade in Endangered Species of Wild Fauna and Flora
D: Digestive
DD: Data deficient
E: Endemic
F: Eye
Fl: Flower
Fr: Fruit
H: Ear
H_2_O_2_: Hydrogen peroxide
HD: Habitats directive annexes
IBA: Internationally important bird areas
ICF: Informant consensus factor
ICPC-2: International Classification of Primary Care, 2nd edition
IL1-α: Interleukin 1-α
IL1-β: Interleukin 1-β
IL-8: Interleukin-8
IPA: Important plant areas
ISE: International Society of Ethnobiology
IUCN: International Union for Conservation of Nature
K: Cardiovascular
L: Musculoskeletal
LC: Least Concern
Le: Leaf
LPS: Lipopolysaccharide
N: Neurological
Ne: Needles
NT: Near threatened
Nt: Number of taxa
Nur: Use reports
P: Psychological
PBA: Primary butterfly areas
ProGEO: International Association for the Conservation of Geological Heritage
R: Respiratory
Re: Resin
Rh: Rhizome
Ro: Root
S: Skin
Se: Seeds
T: Endocrine/metabolic and nutritional
TEK: Traditional ecological knowledge
TNF-α: Tumour necrosis factor alpha
Tu: Tuber
U: Urological
UV: Use values
W: Pregnancy, childbearing, family planning
WFO: World Flora Online
WS: Wild species
WHO: World Health Organization
WhPl: Whole plant
X: Female genital
Y: Male genital
